# Radiosynthesis of (*R*,*S*)‐[^18^F]GE387: A Potential PET Radiotracer for Imaging Translocator Protein 18 kDa (TSPO) with Low Binding Sensitivity to the Human Gene Polymorphism rs6971

**DOI:** 10.1002/cmdc.201900023

**Published:** 2019-04-08

**Authors:** Luxi Qiao, Emily Fisher, Lindsay McMurray, Selena Milicevic Sephton, Matthew Hird, Nisha Kuzhuppilly‐Ramakrishnan, David J. Williamson, Xiouyun Zhou, Eryn Werry, Michael Kassiou, Saijinder Luthra, William Trigg, Franklin I. Aigbirhio

**Affiliations:** ^1^ Molecular Imaging Chemical Laboratory, Wolfson Brain Imaging Centre Department of Clinical Neurosciences University of Cambridge Biomedical Campus Cambridge CB2 0SZ UK; ^2^ School of Chemistry The University of Sydney Building F11, Eastern Avenue Sydney NSW 2006 Australia; ^3^ GE Healthcare Amersham HP7 9LL UK

**Keywords:** [^18^F]GE180, [^18^F]GE387, neuroinflammation biomarkers, PET imaging, TSPO receptor

## Abstract

Translocator protein (TSPO) is a biomarker of neuroinflammation, which is a hallmark of many neurodegenerative diseases and has been exploited as a positron emission tomography (PET) target. Carbon‐11‐labelled PK11195 remains the most applied agent for imaging TSPO, despite its short‐lived isotope and low brain permeability. Second‐generation radiotracers show variance in affinity amongst subjects (low‐, mixed‐, and high‐affinity binders) caused by the genetic polymorphism (rs6971) of the TSPO gene. To overcome these limitations, a new structural scaffold was explored based on the TSPO pharmacophore, and the analogue with a low‐affinity binder/high‐affinity binder (LAB/HAB) ratio similar (1.2 vs. 1.3) to that of (*R*)‐[^11^C]PK11195 was investigated. The synthesis of the reference compound was accomplished in six steps and 9 % overall yield, and the precursor was prepared in eight steps and 8 % overall yield. The chiral separation of the reference and precursor compounds was performed using supercritical fluid chromatography with >95 % *ee*. The absolute configuration was determined by circular dichroism. Optimisation of reaction conditions for manual radiolabelling revealed acetonitrile as a preferred solvent at 100 °C. Automation of this radiolabelling method provided *R* and *S* enantiomers in respective 21.3±16.7 and 25.6±7.1 % decay‐corrected yields and molar activities of 55.8±35.6 and 63.5±39.5 GBq μmol^−1^ (*n*=3). Injection of the racemic analogue into a healthy rat confirmed passage through the blood–brain barrier.

## Introduction

**Figure 1:**
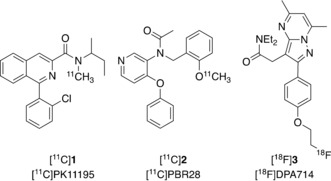
First‐ and second‐generation TSPO PET radiotracers.

Neuroinflammation, an immune response to neuronal insult, is a core component of many disorders such as stroke, multiple sclerosis (MS), neurodegenerative disorders, and brain tumours.^1^ There is a major need to understand the role of neuroinflammation in these pathologies and thereby support the development of therapeutics. One approach for assessing neuroinflammation in vivo is by the use of positron emission tomography (PET) imaging with radiopharmaceuticals that are selective for the 18‐kDa translocator protein (TSPO), a protein with five transmembrane helical domains localised primarily in the outer mitochondrial membrane.^2^, ^3^ Neuroinflammation is driven at least in part by the activation of microglia, the brain's resident macrophages, accompanied by a significant upregulation of TSPO. Therefore, imaging TSPO expression is a powerful approach for investigating neuroinflammation. The most commonly used TSPO PET radiotracer is *R*‐1‐(2‐chlorophenyl)‐*N*‐methyl‐*N*‐(1‐methylpropyl)‐3‐isoquinoline carboxamide ((*R*)‐[^11^C]PK11195, [^11^C]**1**, Figure 1), which is a selective antagonist of TSPO that binds with nanomolar affinity (9.3 nm).^1^, ^4^ (*R*)‐[^11^C]**1** has been used in PET imaging to show neuroinflammation in vivo in cases of stroke, neurodegeneration, traumatic brain injury, and neoplasia.^5^ However, there are a number of challenges that hinder the wider use of [^11^C]**1** in research and clinical settings, such as low brain permeability,^1^, ^4^, ^6^ high nonspecific and variable plasma protein binding, and the short half‐life of carbon‐11 (*t*_1/2_=20 min), which restricts its use to PET centres that have a cyclotron on site.

Given these limitations of [^11^C]**1**, multiple new TSPO radiotracers have been developed and studied; these have improved properties for application, including several that are labelled with the longer‐lived radioisotope fluorine‐18 (*t*_1/2_=109.9 min).^1^ However, PET studies using these second‐generation radiotracers, such as *N*‐(2‐(methoxy‐^11^ *C*)benzyl)‐*N*‐(4‐phenoxypyridin‐3‐yl)acetamide ([^11^C]PBR28, [^11^C]**2**, Figure 1), identified a variance in affinity to these compounds between subjects. This affinity variation was determined to be caused by a genetic polymorphism (rs6971) found in exon 4 of the TSPO gene, which results in substitution of alanine for a threonine at position 147 (A147T). Thus three distinct subject groups have been identified: high‐affinity binders (HABs; Ala/Ala), low‐affinity binders (LABs; Thr/Thr), and mixed‐affinity binders (MABs; (Ala/Thr).^1^

Although previous research has yielded many new radiotracers for imaging TSPO, to date only [^11^C]**1** binds highly independent of TSPO gene polymorphism (e.g., it binds to both LAB and HAB binding sites equally, as indicated by the LAB/HAB ratio of **1**, Table 2), thus prompting further research into the development of an optimised TSPO PET radiotracer, which will ideally have the improved pharmacokinetic properties of second‐generation TSPO radiotracers and be independent of the observed polymorphism.

**Figure 2:**
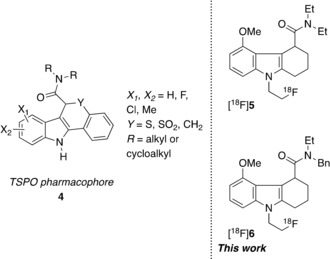
Pharmacophore‐derived second‐generation analogues.

In 2004, a study by Okubo et al. described a tetracyclic indole‐based pharmacophore (**4**, Figure 2) with high affinity for TSPO.^7^ Based on this structural scaffold, a tricyclic derivative [^18^F]GE180 ([^18^F]**5**, Figure 2) was developed with good affinity for TSPO. From radiotracer retention, biodistribution, and in vivo metabolic profile studies, [^18^F]**5** showed high specific binding, good brain uptake, high uptake and retention in the olfactory bulb (TSPO‐enriched region), and good clearance from striatum (region of low TSPO expression) in rats.^8^ This radiotracer has now been evaluated in humans for imaging TSPO expression.^9^, ^10^, ^11^, ^12^

However, while [^18^F]**5** demonstrated less dependence than other second‐generation tracers on the single nucleotide polymorphism, its affinity for TSPO still differs significantly amongst the three binding groups (binding affinity ratio LABs/HABs=5.1).^13^ Therefore, there is still a need to develop a TSPO radiotracer with low sensitivity toward this polymorphism.

Toward this objective informed by structure–activity design features, a novel series of compounds based on the chemical scaffold^14^ of **5** ((*S*)‐GE180) were developed and synthesised. A key design feature being that while retaining good affinity for TSPO, variation at the amide position could influence the LAB/HAB ratio. From this, the compound with most appropriate properties for development as a potential TSPO PET radiotracer with low binding sensitivity to the human gene polymorphism rs6971 was identified as **6** (GE387), the enantiomers of which were shown to have affinities of 1.04 nm (which, later in this work we identified to be the *S* form, as outlined in the Results and Discussion section below) and 22 nm (identified to be the *R* form)^14^ based on a rat heart TSPO assay.^15^ Herein we report, for compound **6**, determination of relative binding affinities to confirm low sensitivity to the genetic polymorphism, synthesis of reference compound **6** and precursors for radiolabelling with fluorine‐18, separation and identification of enantiomers, and finally the development of protocols for the automated radiosynthesis of its radiolabelled analogue [^18^F]**6** as well as the initial in vivo preclinical PET.

## Results and Discussion

### Chemistry

**Scheme 1:**
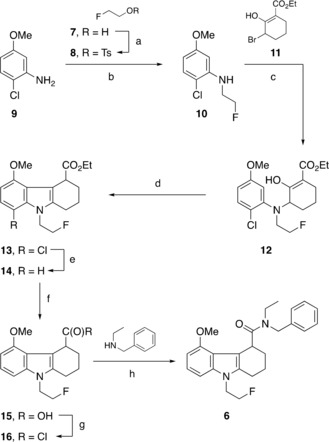
Synthesis of reference compound **6**: a) TsCl, pyridine, 23 °C, 18 h, 26 %; b) **8**, NaH, DMF, 100 °C, 18 h, 32 %; c) KHMDS, THF, 23 °C, 4 h; d) ZnCl_2_, Et_2_O, reflux, 16 h, 31 %; e) H_2_, Pd/C, EtOH, 23 °C, 18 h; f) NaOH, H_2_O, EtOH, reflux, 18 h; g) (COCl)_2_, DMF, 23 °C, 2 h; h) CH_2_Cl_2_, 23 °C, 18 h, 28 % (over four steps).

**Scheme 2:**
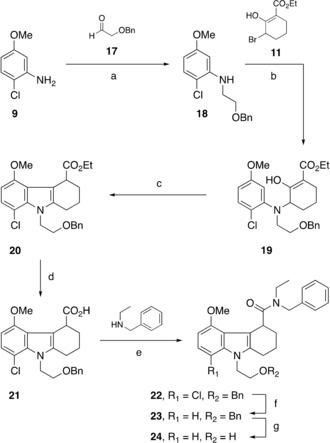
Syntheses of radiolabelling precursors **25** and **26**: a) NaBH(OAc)_3_, CH_2_Cl_2_, 23 °C, 18 h, 74 %; b) KHMDS, THF, 23 °C, 1.5 h; c) ZnCl_2_, Et_2_O, reflux, 5 days, 85 % (over two steps); d) NaOH, H_2_O, EtOH, 80 °C, 18 h; e) HOBt, EDCI, 23 °C, 48 h; f) H_2_, Pd/C, MeOH, Et_3_N, 23 °C, 18 h; g) H_2_, Pd/C, MeOH, 23 °C, 18 h.

**Scheme 3:**
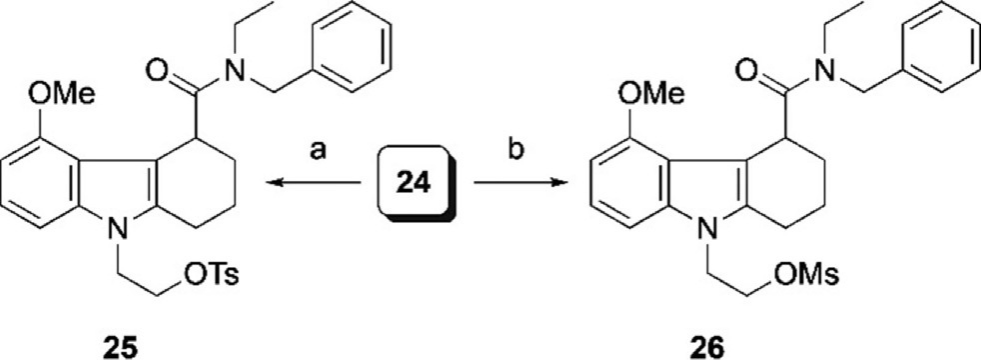
Completion of the syntheses of radiolabelling precursors **25** and **26**: a) TsCl, DMAP, pyridine, 23 °C, 18 h, 13 % (over five steps); b) MsCl, Et_3_N, CH_2_Cl_2_, 23 °C, 18 h, 12 % (over five steps).

For the radiolabelling of [^18^F]**6**, we proposed an S_N_2 nucleophilic substitution of a good leaving group with [^18^F]fluoride, for which reason tosylate **25** and mesylate **26** were envisioned as potential precursors. Reference compound **6** (Scheme 1) and the respective radiolabelling precursors **25** and **26** (Schemes 2 and 3) were prepared by following previously reported syntheses of reference compound and mesylate precursors of **6**.^16^

2‐Fluoroethanol **7** was tosylated in pyridine to form 2‐fluoroethyl tosylate **8**. Tosylate **8** was used to alkylate 2‐chloro‐5‐methoxyaniline **9** to afford **10** (Scheme 1). The methoxyaniline **10** was then deprotonated with KHMDS in THF and reacted with bromide intermediate **11** to give enol **12**, which subsequently underwent cyclisation assisted with zinc chloride in diethyl ether at reflux to afford the tricyclic product **13**, isolated in 31 % yield over two steps. Chloro analogue **13** was dehalogenated with hydrogen on activated palladium to obtain ester **14**, which was hydrolyzed to acid **15**. The crude acid **15** was reacted with *N*‐ethylbenzylamine via an acid chloride intermediate **16** formed with oxalyl chloride to form the reference standard **6** with a yield of 28 % over four steps.

Analogously, 2‐chloro‐5‐methoxyaniline hydrochloride **9** reacted with benzyloxyacetaldehyde **17** under reductive conditions to form the amine **18** (Scheme 2) in 74 % yield. As with the synthesis of the reference compound **6**, the next alkylation step used the same previously made bromide intermediate **11** with KHMDS to yield **19**. The crude product was similarly cyclised using zinc chloride in diethyl ether, to give **20** with 85 % yield. Ester **20** was then hydrolysed using sodium hydroxide in ethanol and water to give acid **21**. Although the two‐step amide formation via the acid chloride intermediate was successfully used in the synthesis of the reference compound **6**, here we found that a one‐step HOBt/EDCI amide coupling was more efficient for the synthesis, yielding **22**. The reaction utilises two activated ester derivatives as intermediates, regenerating HOBt and giving urea as a byproduct. However, on a larger scale this route was not so efficient due to formation of homocoupled byproducts. Compound **22** was dehalogenated in the presence of H_2_ on Pd/C under basic conditions (triethylamine) to give product **23**. A second hydrogenation on crude **23** in the absence of triethylamine was performed to remove the benzyl protecting group to give crude **24** in quantitative yield. Using crude alcohol **24**, we prepared both crude tosylate **25** and crude mesylate precursor **26** (Scheme 3) in a reaction with tosyl or mesyl chloride, with 13 and 12 % yield over five steps, respectively. Tosyl analogue **25** was successfully purified by recrystallisation from EtOAc to give a crystalline solid (33 %). However, the mesyl precursor **26** required purification by column chromatography, yielding a viscous oil (37 %), thus making tosyl precursor **25** practically easier to purify and handle than the mesyl precursor **26**.

**Scheme 4:**
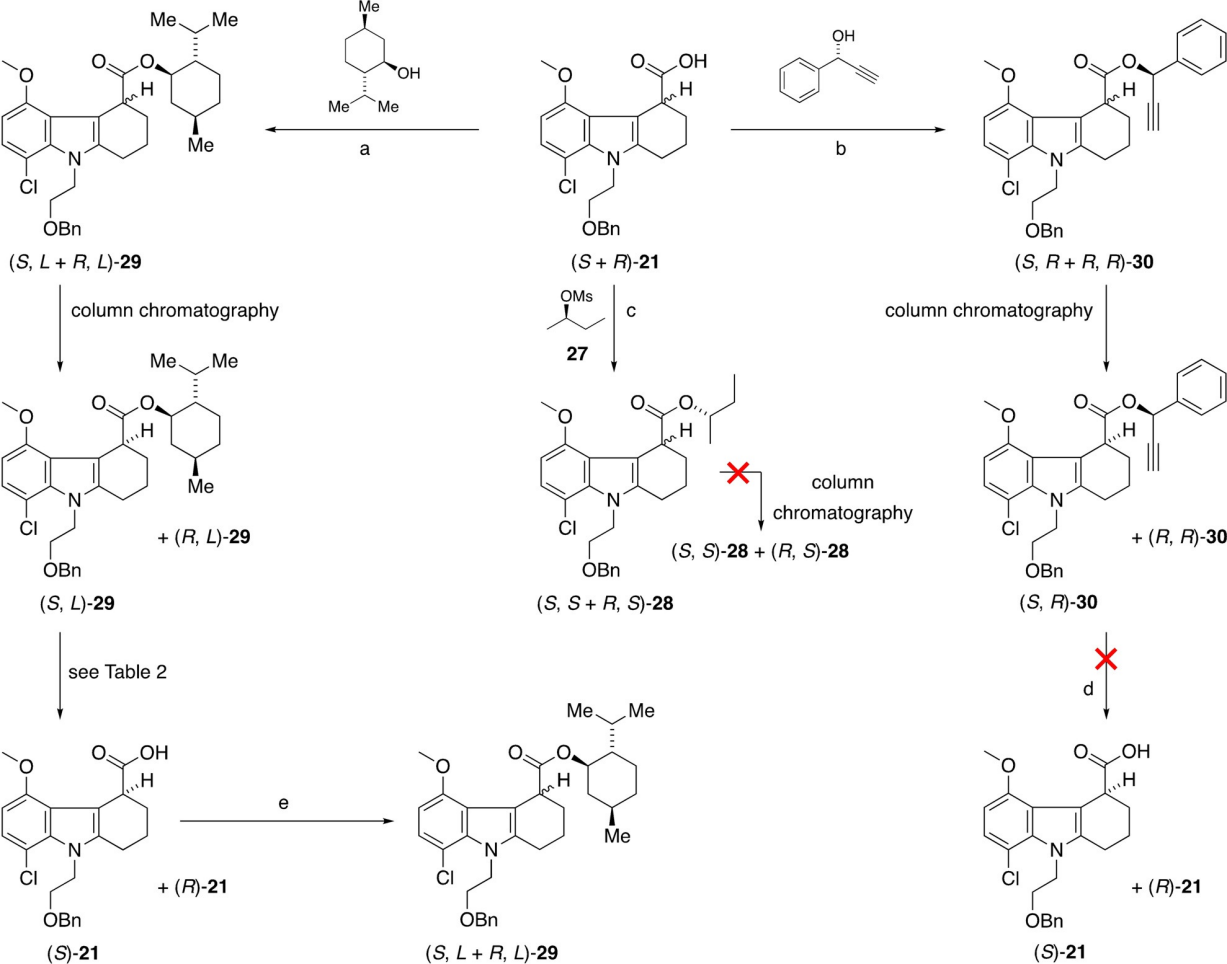
Separation of enantiomers using derivatisation method with chiral alcohols: a) EDCI, DMAP, CH_2_Cl_2_, 23 °C, 16 h, 54 %; b) EDCI, DMAP, CH_2_Cl_2_, 23 °C, 4 h, 31 %; c) DBU, toluene, 80 °C, 4 h; d) EDCI, DMAP, CH_2_Cl_2_, 23 °C, 16 h, 54 %; e) CuCl, MeOH, 40 °C, 6 h.

Because it is well known that enantiomers may have different pharmacokinetic, pharmacodynamic, and safety/toxicity profiles, we also aimed to investigate the difference between the two enantiomers of [^18^F]**6** by establishing the chiral separation of both reference compound **6** as well as its radiolabelling precursor (**25**). A clear choice for chiral separation was found in the preparation of diastereomeric mixture by application of a chiral derivatisation group. We identified an acid intermediate **21** for further functionalisation with several potential enantiomerically pure alcohols. The alcohols selected were l‐menthol, (*R*)‐(−)‐2‐butanol, and (*R*)‐1‐phenyl‐2‐propyn‐1‐ol. The ester **28** with (*R*)‐(−)‐2‐butanol was formed in 53 % yield using DBU as a base and a mesylate analogue **27** of the alcohol as an intermediate. However, a higher‐yielding and a more direct method of EDCI/DMAP coupling was used to form the esters with l‐menthol and (*R*)‐1‐phenyl‐2‐propyn‐1‐ol, which afforded **29** and **30** in 54 and 31 % yields, respectively (Scheme 4).

While NMR analysis demonstrated the presence of two diastereomers in all three product mixtures, TLC analysis of the ester mixtures using a variety of solvent systems showed the two diastereomers could be separated readily by flash column chromatography in two cases, that of l‐menthol and (*R*)‐1‐phenyl‐2‐propynyl analogues. Flash chromatography using diethyl ether and cyclohexane yielded two diasteriomerically enriched samples with both esters **29** (in 78 and 40 % diastereomeric excess) and **30** (in 82 and 35 % diastereomeric excess) (Scheme 4; (*S*,*L*)‐**29**, (*R*,*L*)‐**29** and (*S*,*R*)‐**30**, (*R*,*R*)‐**30**). To demonstrate the presence of a single enantiomer in a mixture, the chiral l‐menthol functionality was first removed under a variety of basic conditions (Table 1). LC–MS analysis showed near‐complete transformation with NaOH/H_2_O in EtOH (entry 1), which were the hydrolytic conditions used in the synthesis of the reference compound **6** and precursors **25** and **26**. Reaction with LiOH in a mixture of THF/H_2_O (entry 2) gave a minor byproduct.^17^

**Table 1:** Conditions for the removal of chiral functionality.

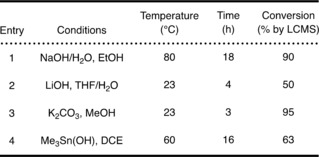

Using K_2_CO_3_ in MeOH (entry 3) gave an equal amount of the desired product and an unidentified byproduct,^18^ while reaction with trimethyltin hydroxide (entry 4) showed an incomplete reaction (63 % by LC) even when the reaction time was increased.^19^ To assess the enantiomeric purity of the product formed, diastereomerically enriched acid was re‐esterified with l‐menthol using EDCI/DMAP. To our disappointment, ^1^H NMR analysis indicated complete racemisation from all investigated hydrolytic reaction conditions. This was further confirmed by chiral HPLC analysis of the acid samples obtained from hydrolysis of diastereomerically enriched ester samples (e.g., (*S*)‐**21**, Scheme 4). Poor stability of the acid **21** in a solution presumably due to the presence of a highly acidic proton is a likely explanation for such rapid racemisation of the stereocenter. In the case of the ester analogue **30**, while we were able to obtain diastereomerically enriched samples through multiple rounds of flash column chromatography in diastereomeric excess ((*S*,*R*)‐**30**, (*R*,*R*)‐**30**), a previously described CuCl hydrolysis protocol failed to give the enantiomerically enriched product **21** (Scheme 3).^20^ Faced with the immediate need to have reference material and precursor separated into enantiomers, we turned to a commercial partner who performed enantiomer separation successfully using SFC for both reference compound **6** and tosylate **25** (see the Supporting Information for details).

### Absolute stereochemistry of enantiomers

**Figure 3:**
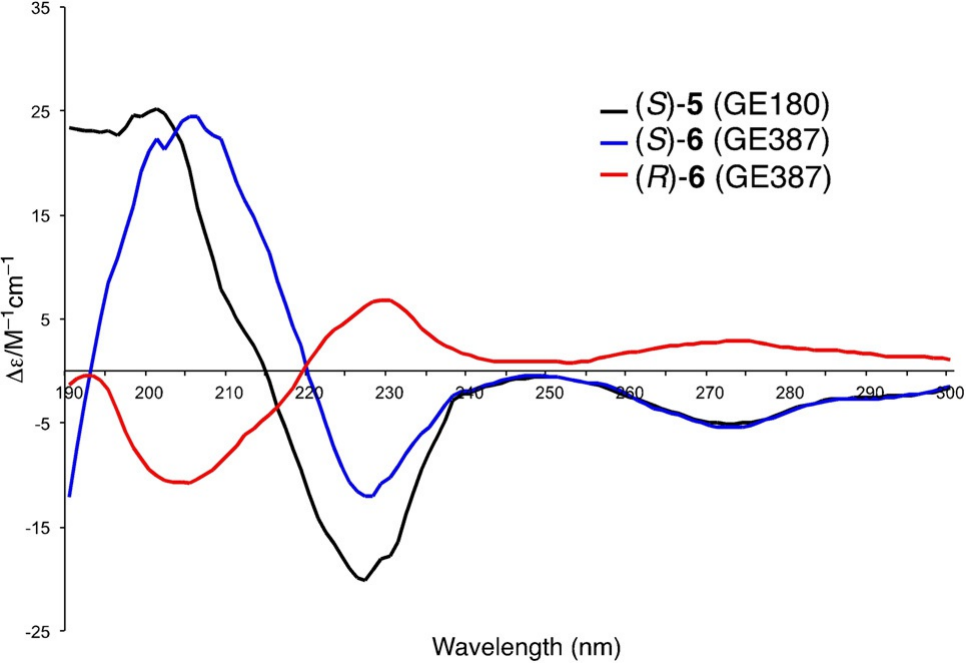
CD spectral data for (*R*)‐**6** and (*S*)‐**6** and comparison with the known CD spectrum of (*S*)‐**5**.

The absolute stereochemistry of enantiomers of **6** was next determined by electronic circular dichroism (CD) spectroscopy whereby CD spectra of **6** were compared with that of **5**, for which stereochemistry had already been established (Figure 3). The spectrum of known (*S*)‐**5** was composed of a maximum positive Cotton effect at 200 nm and a corresponding maximum negative Cotton effect at 226 nm. The enantiomer of **6**, which similarly exhibited a maximum positive Cotton effect at 204 nm and a corresponding maximum negative Cotton effect at 228 nm, was in analogy assigned as the *S* isomer. The other isomer showed the mirror image bisignate band in the CD spectrum and was therefore identified as *R* enantiomer.^21^

To correlate enantiomers obtained via SFC separation to CD data, the chiral HPLC separation was performed on a Whelk‐O1 column to unambiguously identify first eluting peak as the *S* enantiomer (Supporting Information).

### Assessment of GE387 binding affinity to Ala147Thr TSPO

Using an assay based on human embryonic kidney cell lines stably overexpressing human TSPO wild‐type and TSPO A147T^13^ compound **5** was shown to bind to wild‐type TSPO with a higher affinity than compound **1**, but lost affinity at A147T TSPO (Table 2). Compound (*S*)‐**6** bound to wild‐type TSPO with a similar affinity to compound **1**, and both compounds **1** and (*S*)‐**6** only showed minor loss in affinity at A147T TSPO (1.2–1.3× reduction; Table 2). It has been shown for this assay that compounds have lower binding affinities than those commonly reported at rat TSPO. This is also shown for (*S*)‐**6**: 36.3 nm, while 1.04 nm for rat TSPO.^14^

**Table 2:** Binding affinities (*K*_i_) for wild‐type and A147T TSPO.

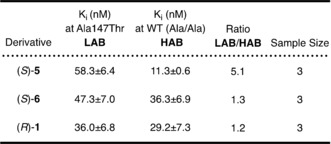

### Radiochemistry

To establish suitable reaction conditions for radiolabelling of [^18^F]**5**, manual radiochemical reactions were performed first. Both precursors **25** and **26** were assessed and the effect of temperature, solvent, and time was investigated (Table 3). For all reactions using **25** (entries 1–6), a single radiolabelled product formed which was identified as [^18^F]**6** via co‐injection of reference compound **6**. However, when using **26** (entries 7–12), a second unidentified radiolabelled product was formed during the reaction. The amount of this radiolabelled side product which formed immediately, did not change during the course of the reaction.

**Table 3:** Screened reaction conditions for radiolabelling of [^18^F]**6**.

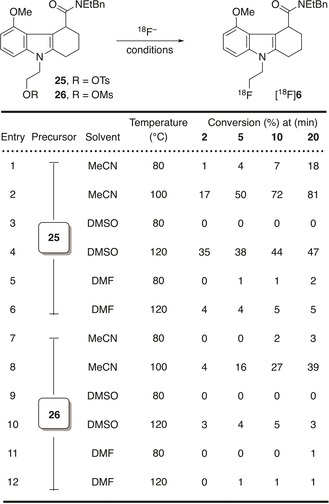

At 80 °C, independent of solvent and precursor, low conversion of <20 % of [^18^F]fluoride to [^18^F]**6** was observed: the highest being 18 % when MeCN was used as a solvent and mesylate **25** as a precursor. When the temperature was increased to 100 °C or 120 °C reactions performed in DMF similarly showed poor conversion (entries 6 and 12) after 20 min. In DMSO, [^18^F]**6** formed with 3 % conversion after 20 min (entry 10) when **26** was used as precursor, whereas tosylate **25** afforded 47 % conversion into [^18^F]**6** under analogous conditions (entry 4). The highest conversion in the case of both precursors **25** and **26** was observed after 20 min in MeCN: 81 and 39 %, respectively (entries 2 and 8).

With this result in hand (MeCN as a solvent, 100 °C, 20 min), next tosylate **25** was used for automated radiolabelling on a GE Healthcare FX_FN_ TRACERlab instrument. [^18^F]Fluorination was achieved with cyclotron‐produced [^18^F]fluoride in MeCN at 100 °C for 20 min. Product [^18^F]**6** was purified by semipreparative reversed‐phase HPLC.

Total radiosynthesis time for the preparation for [^18^F]**6** was 60 min from end of bombardment (EOB). Radiochemical purity was over 99 %, and radiolabelling yield was 20.8±4.5 % decay‐corrected to EOB or 13.6±2.8 % non‐decay‐corrected, and molar radioactivity 93.2±50.6 GBq μmol^−1^ (*n*=9) at the end of synthesis (EOS). Furthermore, the same automated protocol was used for the radiosyntheses of (*R*)‐[^18^F]**6** and (*S*)‐[^18^F]**6** as follows: 21.3±16.7 and 25.6±7.1 % decay‐corrected yields, radiochemical purity >98 % and molar radioactivities 55.8±35.6 and 63.5±39.5 GBq μmol^−1^ (*n*=3) at the EOS, respectively.

### In vivo PET analysis

**Figure 4:**
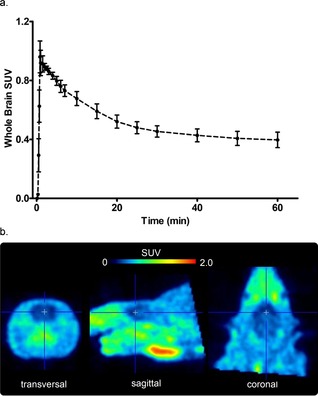
a) Time–activity curve (TAC, *n*=2) over the acquisition time of 60 min and b) summed 30–60 min PET image of racemic [^18^F]**6** in a healthy rat.

As a proof‐of‐principle of our novel radiotracer candidate we next performed PET scans in male Wistar rats to establish that racemic [^18^F]**6** enters the brain (Figure 4). The time–activity curve indicates distribution of radioactivity in the brain, albeit in a modest amount as the investigated animals were healthy.

## Conclusions

Herein we investigated the synthesis of a novel TSPO radiotracer [^18^F]**6**, which has high binding affinity for TSPO. Critically we have established that compound **6** has low sensitivity to the human gene polymorphism rs6971. Based on the synthetic sequence of an analogue [^18^F]**5**, we synthesised the racemate of reference compound **6** and appropriate radiolabelling precursors **25** and **26** for radiolabelling with fluorine‐18. We then explored chiral resolution using the formation of diastereomeric mixtures, but were met with disappointing results. With the racemic precursors in hand, we then optimised radiolabelling conditions under manual reaction conditions and subsequently established the automatic radiolabelling conditions to form both racemic and enantiomerically pure isomers of [^18^F]**6**. The PET scans performed indicate that racemic [^18^F]**6** enters the brain. Detailed studies of biological evaluation of each of the enantiomers of [^18^F]**6** are currently ongoing in our laboratory and will be reported in due course.

## Experimental Section

### General

All reactions requiring anhydrous conditions were conducted in oven‐dried glass apparatus under an atmosphere of inert gas. Reagents were purchased from Sigma–Aldrich or Alfa Aesar. Reagents were used without further purification unless otherwise noted. Triethylamine (Et_3_N) was distilled over P_2_O_5_ and stored over KOH. Reported density values are for ambient temperature. Purity of compounds was ≥95 % as determined by analytical HPLC on a Thermo Dionex 3000 HPLC system. Preparative chromatographic separations were performed on Material Harvest silica gel 60 (35–75 μm) and reactions followed by TLC analysis using Sigma–Aldrich silica gel 60 plates (2–25 μm) with fluorescent indicator (254 nm) and visualised with UV or potassium permanganate.

^1^H NMR spectra were recorded in Fourier transform mode using a Bruker Avance III 300 MHz FT NMR spectrometer and the data were analysed using TopSpin software. Spectra were obtained from the specified deuterated solvents in 5 mm diameter tubes. Chemical shifts in ppm are quoted relative to residual solvent signals calibrated as follows: CDCl_3_ *δ*_H_ (C*H*Cl_3_)=7.26 ppm, *δ*_C_=77.2 ppm. Multiplicities in the ^1^H NMR spectra are described as: s=singlet, d=doublet, t=triplet, q=quartet, quint.=quintet, s=sextet, h=heptet, m=multiplet, b=broad; coupling constants are reported in Hz.

LC–MS data were obtained using a Waters Acquity‐H/Xevo TQD LC–MS instrument. Column: Acquity UPLC® BEH C_18_ 1.7 μm, 2.1×50 mm; flow rate: 0.6 mL min^−1^, 4 min method, 5–95 % MeCN/H_2_O with 0.1 % formic acid. HRMS data were obtained from the EPSRC Mass Spectrometry Service at the University of Swansea. Ion mass/charge (*m*/*z*) ratios are reported as values in atomic mass units.

The automated radiosynthesis was performed on a GE Healthcare FX_FN_ TRACERlab. Semipreparative purification of radiolabelled material was performed on a Merck‐Hitachi L6200A system equipped with Knauer variable wavelength detector and an Eberline radiation detector using a reversed‐phase column (ACE 160433, C_18_, 5 μm, 100×10 mm) and eluting with 48 % aq. MeCN at a flow rate of 5 mL min^−1^. Analytical HPLC samples were analysed by an Agilent HPLC 1100 system equipped with UV multi‐wavelength detector and Raytest Gabi star radiation detector using reversed‐phase column (Phenomenex Luna 5 μm C_18_ (2) 100 Å, 250×4.6 mm, 675295‐11) and eluting with 0–3 min isocratic 10 % aq. MeCN, 3–5 min gradient 10–80 % aq. MeCN, 6–12 min isocratic 80 % aq. MeCN at a flow rate of 1.5 mL min^−1^.

### Chemistry

**2‐Fluoroethyl 4‐methylbenzenesulfonate (8)**. Commercially available 2‐fluoroethanol **7** (0.141 mL, 153 mg, 2.40 mmol, 1 equiv, *d*=1.09 g cm^−3^) was dissolved in pyridine (2.5 mL) under nitrogen. The solution was stirred at 0 °C and tosyl chloride (1.00 g, 5.25 mmol, 2.2 equiv) added portion‐wise to the solution over a period of 30 min, keeping the temperature below 5 °C. The reaction was stirred at RT for 18 h. The reaction was then quenched by careful addition of ice followed by water (5 mL). The reaction mixture was extracted into EtOAc (10 mL) and washed with water (3×10 mL), 1 m HCl solution (10 mL), 1 m aqueous sodium carbonate (10 mL), and copper sulfate (2×10 mL). The organic layer was washed with brine (10 mL), dried (MgSO_4_) and concentrated in vacuo to give **8** as an oil (136 mg, 0.62 mmol, 26 %). LC–MS: *R*_f_ 1.86 (−ESI) *m*/*z* 311.5 ([*M*+94]^+^); ^1^H NMR (300 MHz, CDCl_3_): *δ*_H_=7.81 (2 H, d, *J*=8 Hz, SO_2_CC*H*CH), 7.36 (2 H, d, *J*=8 Hz, SO_2_CCHC*H*), 4.57 (2 H, ddd, *J*=3 and 50 Hz, OCH_2_C*H*_2_F), 4.26 (2 H, ddd, *J*=4 and 30 Hz, OC*H*_2_CH_2_F), and 2.45 (3 H, s, CC*H*_3_). Characterization data for this compound are in complete agreement with previously published data.^16^

**2‐Chloro‐*N*‐(2‐fluoroethyl)‐5‐methoxyaniline (10)**. Commercially available 2‐chloro‐5‐methoxyaniline hydrochloride **9** (5.00 g, 26 mmol, 1 equiv) was dissolved in DMF (60 mL) and sodium hydride (60 % dispersion in mineral oil, 2.30 g, 57 mmol, 2.2 equiv) was added. The reaction was stirred for 30 min at RT under nitrogen. The tosylate **8** (6.00 g, 31 mmol, 1.2 equiv) in DMF was then added dropwise and the reaction was stirred at RT for 2 h. The reaction was then heated at 100 °C for 18 h. The reaction was allowed to cool and the solvent was removed under reduced pressure. The residue was dissolved in EtOAc (100 mL) and washed with water (5×100 mL). The organic layers were combined, dried (MgSO_4_) and concentrated in vacuo to give a brown oil. The crude mixture was then purified by silica flash chromatography eluting with a gradient of 5–30 % EtOAc/petroleum ether (40–60 °C) to yield **10** as a yellow oil (1.69 g, 8.32 mmol, 32 %). LC–MS: *R*_f_ 2.01 (+ESI) *m*/*z* 204.3 ([*M*+H]^+^); ^1^H NMR (300 MHz, CDCl_3_): *δ*_H_=7.16 (1 H, dm, *J*=5 Hz, C*H*C(OCH_3_)CHCH), 6.25–6.22 (2 H, m, CHC(OCH_3_)C*H*C*H*), 4.64 (2 H, dt, *J*=5 and 47 Hz, NCH_2_C*H*_2_F), 4.65–4.55 (1 H, b, NH), 3.77 (3 H, s, OC*H*_3_), and 3.48 (2 H, dq, *J*=5 and 26 Hz, NC*H*_2_CH_2_F). Characterization data for this compound are in complete agreement with an internal GE publication.^14^

**Ethyl 3‐bromo‐2‐hydroxycyclohex‐1‐ene‐1‐carboxylate (11)**. Ethyl 2‐oxocyclohexanecarboxylate **S1** was dissolved in diethyl ether and cooled to 0 °C under nitrogen. Bromine was added dropwise over 15 min and the reaction mixture was allowed to warm to RT over 90 min. The mixture was slowly poured into ice‐cold saturated aqueous potassium carbonate and extracted with ethyl acetate. The combined organic layers were dried over magnesium sulfate, filtered, concentrated in vacuo and dried on the vacuum line for 18 h to afford **11** as a yellow oil. LC–MS: *R*_f_ 2.17; ^1^H NMR (300 MHz, CDCl_3_): *δ*_H_=11.92 (1 H, s, O*H*), 4.93 (1 H, t, *J*=3.4 Hz, BrC*H*C(OH)), 4.20 (2 H, ddd, *J*=1, 7 and 14 Hz, C(O)OC*H*_2_CH_3_), 2.53–2.46 (2 H, m, BrCHCH_2_CH_2_C*H*_2_), 2.12–2.03 (2 H, m, BrCHC*H*_2_CH_2_CH_2_), 1.79–1.67 (2 H, m, BrCHCH_2_C*H*_2_CH_2_), 1.24 (3 H, t, *J*=7 Hz, C(O)OCH_2_C*H*_3_). Characterization data for this compound are in complete agreement with an internal GE publication.^14^

**Ethyl‐3‐((2‐chloro‐5‐methoxyphenyl)(2‐fluoroethyl)amino)‐2‐hydroxycyclohex‐1‐ene‐1‐carboxylate (12)**. A solution of the aniline **10** (2.20 g, 10.8 mmol, 1 equiv) in THF (60 mL) was cooled to −40 °C. Potassium bis(trimethylsilyl)amide (0.5 m solution in toluene, 45.4 mL, 4.5 g, 22.7 mmol, 2.1 equiv) was added dropwise and the reaction stirred for 30 min at −40 °C. The carboxylate **11** (2.67 g, 10.8 mmol, 1 equiv) in THF (10 mL) was added dropwise at −40 °C. The cooling bath was removed and the reaction was stirred at RT for 4 h. The reaction was quenched with brine (100 mL) and extracted into EtOAc (2×200 mL), dried (MgSO_4_) and concentrated in vacuo to give **12** as a brown oil (3.70 g, 9.97 mmol) which was used crude in the next step. LC–MS: *R*_f_ 2.29 (+ESI) *m*/*z* 372.4 ([*M*+H]^+^). Characterization data for this compound are in complete agreement with an internal GE publication.^14^

**Ethyl 8‐chloro‐9‐(2‐fluoroethyl)‐5‐methoxy‐2,3,4,9‐tetrahydro‐1*H*‐carbazole‐4‐carboxylate (13)**. The intermediate **12** (3.71 g, 10.0 mmol, 1 equiv) was dissolved in Et_2_O (100 mL) and zinc chloride (5.45 g, 40.0 mmol, 4 equiv) was added. The reaction was heated at reflux for 16 h. EtOAc (300 mL) was added to dissolve everything and was washed with 2 m HCl (200 mL), water (200 mL), 10 % aqueous potassium carbonate (200 mL), dried (MgSO_4_) and concentrated in vacuo. The crude material was purified by silica gel chromatography eluting with a gradient of 5–20 % of EtOAc/petroleum ether (40–60 °C) to afford **13** as a yellow oil (1.1 g, 3.12 mmol, 31 % over two steps). Material could not be obtained in a pure form, but regardless, the reaction was progressed to the next step. LC–MS: *R*_f_ 2.38 (+ESI) *m*/*z* 354.4 ([*M*+H]^+^); ^1^H NMR (300 MHz, CDCl_3_): *δ*_H_=6.95 (1 H, d, *J*=8 Hz, 7‐C*H*), 6.35 (1 H, d, *J*=8 Hz, 6‐C*H*), 4.90–4.40 (4 H, m, NC*H*_2_CH_2_F, NCH_2_C*H*_2_F), 4.20–4.10 (3 H, m, 4‐C*H*, CO_2_C*H*_2_CH_3_), 2.80–2.65 (2 H, m, 1‐C*H*_2_), 3.79 (3 H, s, OC*H*_3_), 2.10–1.80 (4 H, m, 2‐ and 3‐C*H*_2_), and 1.30–1.20 (3 H, m, CO_2_CH_2_C*H*_3_). Characterization data for this compound are in complete agreement with an internal GE publication.^14^

**Ethyl 9‐(2‐fluoroethyl)‐5‐methoxy‐2,3,4,9‐tetrahydro‐1*H*‐carbazole‐4‐carboxylate (14)**. The chloro intermediate **13** (1.10 g, 3.11 mmol, 1 equiv) was dissolved in methanol (50 mL) and triethylamine (0.517 mL, 377 mg, 3.73 mmol, 1.2 equiv, *d*=0.73 g cm^−3^) and 10 % Pd/C (0.414 g) were added. The mixture was stirred for 18 h after purging twice (over 2 h) with hydrogen gas under atmospheric pressure. The reaction was filtered through a pad of Celite under nitrogen atmosphere, washed with methanol (50 mL), and the solvent was removed in vacuo. The residue was dissolved in EtOAc (100 mL) and washed with 10 % aqueous potassium carbonate (100 mL), dried (MgSO_4_) and concentrated in vacuo to give **14** as an oil (0.75 g, 2.35 mmol, 76 %). LC–MS: *R*_f_ 2.18 (+ESI) *m*/*z* 320.5 ([*M*+H]^+^, 43 %), *R*_f_ 2.20 (−ESI) *m*/*z* 318.4 ([*M*−H]^−^); ^1^H NMR (300 MHz, CDCl_3_): *δ*_H_=7.04 (1 H, t, *J*=8 Hz, 7‐C*H*), 6.84 (1 H, d, *J*=8 Hz, 8‐C*H*), 6.46 (1 H, d, *J*=8 Hz, 6‐C*H*), 4.64 (2 H, dm, *J*=47 Hz, NCH_2_C*H*_2_F), 4.40–4.00 (5 H, m, 4‐C*H*, CO_2_C*H*_2_CH_3_, NC*H*_2_CH_2_F), 3.82 (3 H, s, OC*H*_3_), 2.90–2.60 (2 H, m, 1‐C*H*_2_), 2.20–1.80 (4 H, m, 2‐ and 3‐C*H*_2_), and 1.30–1.20 (3 H, m, CO_2_CH_2_C*H*_3_). Characterization data for this compound are in complete agreement with an internal GE publication.^14^

**9‐(2‐Fluoroethyl)‐5‐methoxy‐2,3,4,9‐tetrahydro‐1*H*‐carbazole‐4‐carboxylic acid (15)**. The ester **14** (0.747 g, 2.34 mmol, 1 equiv) was dissolved in ethanol (10 mL). A solution of sodium hydroxide (1.13 g, 28.2 mmol, 12 equiv) dissolved in 10 mL of water, was added. The reaction mixture was heated at reflux for 18 h. The solvent was removed in vacuo and the crude mixture diluted with water (30 mL), acidified with 2 m HCl dropwise until acidic, and washed with CH_2_Cl_2_ (50 mL). The organic layers were combined and dried (MgSO_4_) and concentrated in vacuo to give **15** (0.66 g, 2.27 mmol), which was used crude into the next step. LC–MS: *R*_f_ 1.81 (+ESI) *m*/*z* 292.4 ([*M*+H]^+^).

**9‐(2‐Fluoroethyl)‐5‐methoxy‐2,3,4,9‐tetrahydro‐1*H*‐carbazole‐4‐carbonyl chloride (16)**. A solution of **15** (0.660 g, 2.27 mmol, 1 equiv) in anhydrous CH_2_Cl_2_ (5 mL) was stirred under nitrogen. Oxalyl chloride (0.584 mL, 865 mg, 6.81 mmol, 3 equiv, *d*=1.48 g cm^−3^) was added followed by a drop of DMF. The reaction mixture was stirred at RT under nitrogen for 2 h then evaporated in vacuo to give **16** (1.03 g, 3.33 mmol) as an oil, which was used into the next step without purification.

***N*** **‐Benzyl‐*N*‐ethyl‐9‐(2‐fluoroethyl)‐5‐methoxy‐2,3,4,9‐tetrahydro‐1*H*‐carbazole‐4‐carboxamide (GE387) (6)**. The acid chloride **16** (1.03 g, 3.33 mmol, 1 equiv) was dissolved in anhydrous CH_2_Cl_2_ (8 mL) and cooled to 0 °C. *N*‐Ethylbenzylamine (0.977 mL, 901 mg, 6.67 mmol, 2 equiv, *d*=0.92 g cm^−3^) was then added and the reaction was stirred for 18 h at RT. The reaction was quenched with 10 % aqueous potassium carbonate (6 mL). The CH_2_Cl_2_ layer was collected via a separatory funnel, dried (MgSO_4_), and then concentrated in vacuo. The crude material was purified by silica gel chromatography eluting with a gradient of 50–100 % EtOAc/petroleum ether (40–60 °C) to afford the crude product as a yellow oil. The oil was then recrystallised from EtOAc to afford **6** as a pale‐yellow solid (350 mg, 0.86 mmol, 26 % over two steps). LC–MS: *R*_f_ 2.30 (+ESI) *m*/*z* 409.6 ([*M*+H]^+^, 31 %), *R*_f_ 2.35 (+ESI) *m*/*z* 409.6 ([*M*+H]^+^); ^1^H NMR (300 MHz, CDCl_3_): *δ*_H_=7.42–7.21 (5 H, m, Ph), 7.02 (1 H, t, *J*=8 Hz, 7‐CH), 6.84 (1 H, d, *J*=8 Hz, 8‐CH), 6.43 (1 H, d, *J*=8 Hz, 7‐CH), 4.80–4.40 (3 H, m, CH_2_C*H*_2_F, 4‐C*H*), 4.31 (2 H, dt, *J*=5 and 24 Hz NC*H*_2_CH_2_F), 3.62 (3 H, s, OC*H*_3_), 3.83–3.41 (4 H, m, N(C*H*_2_CH_3_)_2_), 2.88–2.61 (2 H, m, 1‐C*H*_2_), 2.20–1.80 (4 H, m, 2‐ and 3‐C*H*_2_), and 1.32 (3 H, t, *J*=7 Hz, N(CH_2_C*H*_3_)_2_).

***N*** **‐(2‐(Benzyloxy)ethyl)‐2‐chloro‐5‐methoxyaniline (18)**. Commercially available **9** (11.7 g, 60.5 mmol, 1 equiv) was converted into the free base with 1 m aqueous sodium carbonate (300 mL). The mixture was extracted with CH_2_Cl_2_ (2×200 mL), the organic layer dried (MgSO_4_) and evaporated to give an oil. Compound **9** was then dissolved in CH_2_Cl_2_ (50 mL), in a dry flask under nitrogen. Benzyloxyacetaldehyde **17** (9.36 mL, 10.0 g, 66.6 mmol, 1.1 equiv, *d*=1.07 g cm^−3^) and acetic acid (3.81 mL, 4.0 g, 66.6 mmol, 1.1 equiv, *d*=1.05 g cm^−3^) were added. After 15 min sodium triacetoxyborohydride (19.3 g, 90.8 mmol, 1.5 equiv) was added. The mixture was stirred for 18 h at RT and then poured into saturated aqueous ammonium chloride solution (100 mL) and extracted with CH_2_Cl_2_ (2×100 mL). The combined organic layers were dried (MgSO_4_) and evaporated to give an oil. The crude product was purified via silica flash chromatography eluting with CH_2_Cl_2_ to yield **18** as an oil (13.0 g, 44.8 mmol, 74 %). Material could not be obtained in a pure form, but regardless, the reaction was progressed to the next step. LC–MS: *R*_f_ 2.42 (+ESI) *m*/*z* 292.3 ([*M*+H]^+^); ^1^H NMR (300 MHz, CDCl_3_): *δ*_H_=7.37–7.30 (5 H, m, Ph), 7.14 (1 H, d, *J*=8 Hz, C*H*C(OCH_3_)CHCH), 6.23–6.18 (2 H, m, CHC(OCH_3_)C*H*C*H*), 4.71 (2 H, s, OC*H*_2_Ph), 3.74 (3 H, s, OC*H*_3_), 3.73 (2 H, t, *J*=5 Hz, C*H*_2_O), and 3.35 (2 H, dd, *J*=6 Hz, C*H*_2_N). Characterization data for this compound are in complete agreement with an internal GE publication.^14^

**Ethyl 3‐((2‐(benzyloxy)ethyl)(2‐chloro‐5‐methoxyphenyl)amino)‐2‐hydroxycyclohex‐1‐ene‐1‐carboxylate (19)**. The aniline **18** (14.1 g, 48.5 mmol, 1 equiv) was stirred in dry THF (150 mL) at −40 °C under nitrogen and potassium bis(trimethylsilyl) amide (0.5 m solution in toluene, 203 mL, 20.2 g, 101.9 mmol, 2.1 equiv) was added over 30 min. The carboxylate **11** (12.0 g, 48.5 mmol, 1 equiv) in dry THF (50 mL) was then added and allowed to warm to RT over a period of 1.5 h. Acetic acid was added (15 mL) and concentrated in vacuo to remove the THF. EtOAc (200 mL) and 10 % aqueous potassium carbonate (200 mL) was added and the mixture vigorously shaken. The EtOAc solution was separated, dried over (MgSO_4_) and concentrated in vacuo to afford **19** as an oil (24.0 g, 52.2 mmol), which was used crude in the next step. LC–MS: *R*_f_ 2.39 (−ESI) *m*/*z* 458.5 ([*M*−H]^−^), *R*_f_ 2.53 (+ESI) *m*/*z* 460.5 ([*M*+H]^+^), *R*_f_ 2.39 (+ESI) *m*/*z* 460.5 ([*M*+H]^+^). Characterization data for this compound are in complete agreement with an internal GE publication.^14^

**Ethyl 9‐(2‐(benzyloxy)ethyl)‐8‐chloro‐5‐methoxy‐2,3,4,9‐tetrahydro‐1*H*‐carbazole‐4‐carboxylate (20)**. Zinc chloride (19.6 g, 144 mmol, 3 equiv) was added to carboxylate **19** (22.0 g, 48.0 mmol, 1 equiv) in dry Et_2_O (400 mL) under nitrogen and heated at reflux for 5.5 h. As the reaction was held at reflux, a thick brown dense oil formed in the reaction. The reaction was then cooled and the supernatant Et_2_O decanted off, EtOAc (300 mL) was added, washed with 2 m HCl (150 mL) and with 10 % aqueous potassium carbonate (150 mL). The EtOAc layer was separated, dried (MgSO_4_) and concentrated in vacuo to afford an oil. The crude material was purified by silica gel chromatography eluting with a gradient of 10–40 % EtOAc/petroleum ether (40–60 °C) to afford **20** as an oil (1.98 g, 4.49 mmol). The thick dense brown layer was treated with EtOAc (300 mL) and 2 m HCl (150 mL). The EtOAc layer was separated, washed with 10 % aqueous potassium carbonate (150 mL), dried (MgSO_4_) and concentrated in vacuo to give an oil. Et_2_O (400 mL) and anhydrous zinc chloride (19.6 g, 144 mmol, 3 equiv) were added. The mixture was heated at reflux for a further 5 days. The Et_2_O layer and the dark gum were both diluted with EtOAc (200 mL) and then washed with 2 m HCl (150 mL), dried (MgSO_4_) and concentrated in vacuo to give a gum. This gum was purified by silica gel chromatography eluting with a gradient of 5–35 % EtOAc/petroleum ether (40–60 °C) to afford **20** as an oil (16.1 g, 36.5 mmol, 85 % total over two steps). LC–MS: *R*_f_ 2.66 (+ESI) *m*/*z* 442.5 ([*M*+H]^+^); ^1^H NMR (300 MHz, CDCl_3_): *δ*_H_=7.31–7.16 (5 H, m, Ph), 6.93 (1 H, d, *J*=8 Hz, 7‐CH_2_), 6.34 (1 H, d, *J*=8 Hz, 6‐CH_2_), 4.58 (2 H, dm, *J*=62 Hz, NC*H*_2_CH_2_O), 4.40 (2 H, s, OC*H*_2_Ph), 4.20–4.09 (3 H, m, CO_2_C*H*_2_CH_3_ and 4‐CH), 3.86–3.80 (2 H, m, C*H*_2_OBn), 3.79 (3 H, s, OCH_3_), 2.82–2.63 (2 H, m, 1‐CH_2_), 2.10–1.80 (4 H, m, 2‐ and 3‐CH_2_), and 1.23 (3 H, t, *J*=7 Hz, CO_2_CH_2_C*H*_3_). Characterization data for this compound are in complete agreement with an internal GE publication.^14^

**9‐(2‐(Benzyloxy)ethyl)‐8‐chloro‐5‐methoxy‐2,3,4,9‐tetrahydro‐1*H*‐carbazole‐4‐carboxylic acid (21)**. To the ester **20** (0.401 g, 0.909 mmol, 1 equiv) in ethanol (12 mL) was added sodium hydroxide (0.240 g, 6.00 mmol, 6.6 equiv) in water (1 mL) and heated at 80 °C for 18 h. The ethanol was then removed by evaporation in vacuo and the residue partitioned between Et_2_O (30 mL) and water (30 mL). The Et_2_O layer was separated, dried (MgSO_4_) and concentrated in vacuo to give a gum. The aqueous layer was acidified to pH 1 with 2 m HCl dropwise and extracted with CH_2_Cl_2_ (3×20 mL). The organic layers were dried (MgSO_4_) and concentrated in vacuo to afford **21** as a solid (0.36 g, 0.87 mmol, 93 %). LC–MS: *R*_f_ 2.34 (+ESI) *m*/*z* 414.4 ([*M*+H]^+^); ^1^H NMR (300 MHz, CDCl_3_): *δ*_H_=7.31–7.15 (5 H, m, Ph), 6.97 (1 H, d, *J*=8 Hz, 7‐CH), 6.41 (1 H, d, *J*=8 Hz, 6‐CH), 4.68–4.52 (2 H, m, NC*H*_2_CH_2_O), 4.40 (2 H, s, OC*H*_2_Ph), 4.18 (1 H, t, *J*=3 Hz, 4‐CH), 3.88 (3 H, s, OC*H*_3_), 3.82 (2 H, t, *J*=6 Hz, C*H*_2_OBn), 2.86–2.63 (2 H, m, 1‐CH_2_), and 2.27–1.86 (4 H, m, 2‐ and 3‐CH_2_). Characterization data for this compound are in complete agreement with an internal GE publication.^14^

***N*** **‐Benzyl‐9‐(2‐(benzyloxy)ethyl)‐8‐chloro‐*N*‐ethyl‐5‐methoxy‐2,3,4,9‐tetrahydro‐1*H*‐carbazole‐4‐carboxamide (22)**. The acid **21** (2.23 g, 5.40 mmol, 1 equiv) was dissolved in THF (135 mL) under N_2_ at RT. The solution was allowed to cool to 0 °C and *N*‐ethylbenzylamine (0.870 mL, 803 mg, 5.94 mmol, 1.1 equiv, *d*=0.92 g cm^−3^) was added followed by 1‐hydroxybenzotriazole hydrate (0.802 g, 5.94 mmol, 1.1 equiv) and *N*‐(3‐dimethylaminopropyl)‐*N*′‐ethylcarbodiimide hydrochloride (2.07 g, 10.8 mmol, 2 equiv). Finally, triethylamine (2.99 mL, 2.18 g, 10.8 mmol, 4 equiv, *d*=0.73 g cm^−3^) was added via syringe and the mixture stirred under N_2_ and warmed to RT over 48 h. The reaction was then diluted with EtOAc (100 mL) and filtered through Celite. The Celite was washed with more EtOAc (2×100 mL). The combined filtrates were washed with 1 m aqueous HCl (2×150 mL), H_2_O (2×150 mL), brine (150 mL), dried (MgSO_4_) and concentrated in vacuo to give the product **22** as an oil (1.55 g, 2.92 mmol, 54 %), which was used crude in the next step. LC–MS: *R*_f_ 2.74 (+ESI) *m*/*z* 531.6 ([*M*+H]^+^).

***N*** **‐Benzyl‐9‐(2‐(benzyloxy)ethyl)‐*N*‐ethyl‐5‐methoxy‐2,3,4,9‐tetrahydro‐1*H*‐carbazole‐4‐carboxamide (23)**. The carboxamide **22** (1.50 g, 2.83 mmol, 1 equiv) in methanol (120 mL) was shaken with 10 % palladium on charcoal (1.5 g), triethylamine (0.549 mL, 401 mg, 3.96 mmol, 1.4 equiv, *d*=0.73 g cm^−3^) and stirred for 18 h at RT after purging twice (over 2 h) with hydrogen gas under atmospheric pressure. The reaction was then filtered through a pad of Celite under nitrogen atmosphere and the filtrate concentrated in vacuo. The concentrate was then taken up in CH_2_Cl_2_ (200 mL) and washed with 5 % aqueous potassium carbonate solution (200 mL). The CH_2_Cl_2_ solution was then separated, dried (MgSO_4_) and concentrated in vacuo to afford **23** as an oil (1.10 g, 2.22 mmol), which was used crude in the next step. LC–MS: *R*_f_ 2.62 (+ESI) *m*/*z* 497.5 ([*M*+H]^+^).

***N*** **‐Benzyl‐*N*‐ethyl‐9‐(2‐hydroxyethyl)‐5‐methoxy‐2,3,4,9‐tetrahydro‐1*H*‐carbazole‐4‐carboxamide (24)**. The benzyl‐protected intermediate **23** (1.10 g, 2.22 mmol, 1 equiv) in methanol (50 mL) was shaken with 10 % palladium on charcoal (0.333 g) and stirred for 18 h at RT after purging twice (over 2 h) with hydrogen gas under atmospheric pressure. The reaction was then filtered through a pad of Celite under nitrogen atmosphere and the filtrate concentrated in vacuo to give **24** as an oil (900 mg, 2.22 mmol), which was used crude in the next step. LC–MS: *R*_f_ 2.08 (+ESI) *m*/*z* 407.5 ([*M*+H]^+^).

**2‐(4‐(Benzyl(ethyl)carbamoyl)‐5‐methoxy‐1,2,3,4‐tetrahydro‐9*H*‐carbazol‐9‐yl)ethyl4‐methylbenzenesulfonate (25)**. The alcohol **24** (500 mg, 1.23 mmol, 1 equiv) was dissolved in anhydrous pyridine (5 mL) under N_2_. The solution was cooled to 0 °C and 4‐toluenesulfonyl chloride (515 mg, 2.71 mmol, 2.2 equiv) added potion‐wise to the solution over 30 min. 4‐Dimethylaminopyridine (15.0 mg, 0.123 mmol, 0.1 equiv) was then added, and the solution was stirred and warmed to RT for 18 h. The reaction was then quenched by careful addition of ice followed by water (5 mL). The mixture was then extracted into EtOAc and washed with water (3×10 mL). Excess pyridine was removed by washing with 1 m HCl (2×10 mL) and aqueous copper sulfate (2×10 mL). Excess 4‐toluenesulfonyl chloride was removed by washing with 1 m aqueous sodium carbonate (2×10 mL). The organic layer was washed with brine (10 mL), dried (MgSO_4_) and concentrated in vacuo. The crude product was recrystallised from EtOAc to afford **25** as an off‐white solid (220 mg, 0.40 mmol, 33 %). LC–MS: *R*_f_ 2.46 (+ESI) *m*/*z* 561.6 ([*M*+H]^+^); ^1^H NMR (300 MHz, CDCl_3_): *δ*_H_=7.51 ((2 H, d, *J*=8 Hz, SO_2_C(C*H*_2_)(CH_2_)CH_3_), 7.35–7.15 (5 H, m, Ph), 7.09 (2 H, d, *J*=8 Hz, SO_2_C(CH_2_)(C*H*_2_)CH_3_), 6.91 (1 H, t, *J*=8 Hz, 7‐C*H*), 6.62 (1 H, d, *J*=8 Hz, 8‐C*H*), 6.37 (1 H, d, *J*=8 Hz, 6‐C*H*), 4.70–4.50 (2 H, m, C*H*_2_N), 4.30–4.15 (3 H, m, 4‐C*H* and C*H*_2_OH), 3.61 (3 H, s, OC*H*_3_), 3.80–3.40 (4 H, m, N(C*H*_2_)_2_), 2.80–2.50 (2 H, m, 1‐C*H*_2_), 2.33 (3 H, s, SO_2_PhC*H*_3_), 2.1–1.60 (4 H, m, 2‐ and 3‐CH_2_), and 1.30 (3 H, t, *J*=7 Hz, NCH_2_C*H*_3_).

**2‐(4‐(Benzyl(ethyl)carbamoyl)‐5‐methoxy‐1,2,3,4‐tetrahydro‐9*H*‐carbazol‐9‐yl)ethyl methanesulfonate (26)**. The phenol **24** (400 mg, 0.985 mmol, 1 equiv) in CH_2_Cl_2_ (20 mL) was cooled to 0 °C and methanesulfonyl chloride (0.229 mL, 338 mg, 2.95 mmol, 3 equiv, *d*=1.48 g cm^−3^) and triethylamine (0.409 mL, 299 mg, 2.95 mmol, 3 equiv, *d*=0.73 g cm^−3^) were added and allowed to warm to RT for 18 h. The reaction was diluted with CH_2_Cl_2_ (20 mL) washed with 5 % aqueous potassium carbonate solution (40 mL). The layers were separated. The combined organic layers were dried (MgSO_4_) and concentrated in vacuo to give a gum. The crude material was purified by silica gel flash chromatography eluting with a gradient of 50–100 % EtOAc/petroleum ether (40–60 °C) to afford **26** as an oil (142 mg, 0.29 mmol, 37 %). LC–MS: *R*_f_ 2.12 (+ESI) *m*/*z* 485.5 ([*M*+H]^+^). ^1^H NMR (300 MHz, CDCl_3_): *δ*_H_=7.40–7.10 (5 H, m, NCH_2_Ph), 7.03 (1 H, t, *J*=8 Hz, 7‐C*H*), 6.86 (1 H, d, *J*=8 Hz, 8‐C*H*), 6.44 (1 H, d, *J*=8 Hz, 6‐C*H*), 4.65–4.50 (2 H, m, C*H*_2_N), 4.50–4.45 (3 H, m, 4‐C*H* and C*H*_2_OH), 3.64 (3 H, s, OC*H*_3_), 3.90–3.20 (4 H, m, N(C*H*_2_)_2_), 2.90–2.60 (2 H, m, 1‐C*H*_2_), 2.51 (3 H, s, OSO_2_C*H*_3_), 2.30–1.70 (4 H, m, 2‐ and 3‐CH_2_), and 1.31 (3 H, t, *J*=7 Hz, NCH_2_C*H*_3_).

**(1*R*,2*S*,5*R*)‐2‐Isopropyl‐5‐methylcyclohexyl 9‐(2‐(benzyloxy)ethyl)‐8‐chloro‐5‐methoxy‐2,3,4,9‐tetrahydro‐1*H*‐carbazole‐4‐carboxylate (29)**. To a stirring solution of **21** (300 mg, 0.726 mmol, 1 equiv) and l‐menthol in CH_2_Cl_2_ (40 mL) at 0 °C was added *N*‐(3‐Dimethylaminopropyl)‐*N*′‐ethylcarbodiimide hydrochloride (139 mg, 0.726 mmol, 1 equiv) and 4‐Dimethylaminopyridine (92.8 mg, 0.726 mmol, 1 equiv). Stirring was continued at RT for 16 h. After this period, H_2_O (100 mL) and CH_2_Cl_2_ (100 mL) were added. The organic layer was washed with 1 m HCl, saturated NaHCO_3_ solution, and brine. The solution was dried (MgSO_4_), filtered, and concentrated. The crude racemic mixture **29** (360 mg, 0.65 mmol) was separated into two diasteriomerically enriched samples via silica flash chromatography eluting with 30 % Et_2_O/cyclohexane. LC–MS: *R*_f_ 3.12 (+ESI) *m*/*z* 368.3 ([*M*−182]^+^, 100 %); ^1^H NMR (300 MHz, CDCl_3_): *δ*_H_=7.36–7.12 (5 H, m, Ph), 6.94 (1 H, d, *J*=8 Hz, 7‐CH), 6.31 (1 H, d, *J*=8 Hz, 6‐CH), 4.90 (1 H, dt, *J*=4 and 6 Hz, OC*H*), 4.38 (2 H, d, *J*=6 Hz, NC*H*_2_CH_2_O), 4.09 (1 H, t, *J*=6 Hz, 4‐CH), 3.86–3.77 (2 H, m, C*H*_2_OBn), 3.75 (3 H, s, OCH_3_), 2.7–2.44 (3 H, m, 1‐CH_2_, OCHC*H*_2_), 2.15–2.01 (1 H, m, OCHC*H*_2_), 2.00–1.82 (3 H, m, 2‐CH_2_, OCHC*H*), 1.81–1.56 (4 H, m, 3‐CH_2_, CH_3_C*H*, OCHCHC*H*_2_), 1.46–1.40 (12 H, m, CH_3_CHC*H*_2_, OCHCHC*H*_2_, CHC*H*_3_, CH(C*H*_3_)_2_, and 1.02–0.83 (1 H, m, CH_3_CHC*H*_2_).

**(*R*)‐1‐Phenylprop‐2‐yn‐1‐yl 9‐(2‐(benzyloxy)ethyl)‐8‐chloro‐5‐methoxy‐2,3,4,9‐tetrahydro‐1*H*‐carbazole‐4‐carboxylate (30)**. To a solution of **21** (300 mg, 0.73 mmol, 1 equiv), *N*‐(3‐Dimethylaminopropyl)‐*N*′‐ethylcarbodiimide hydrochloride (170 mg, 1.09 mmol, 1.5 equiv) and *4*‐Dimethylaminopyridine (6.96 mg, 0.04 mmol, 0.05 equiv) in CH_2_Cl_2_ (5 mL) was added (*R*)‐*1*‐phenyl‐*2*‐propyn‐*1*‐ol (0.09 mL, 95.9 mg, 0.726 mmol, 1 equiv, *d*=1.07 g cm^−3^) at RT. The reaction was stirred for 4 h at RT. The reaction mixture was diluted with CH_2_Cl_2_ (20 mL) and washed with H_2_O (5 mL). The organic layer was dried (MgSO_4_) and concentrated. The crude mixture was purified via silica flash column chromatography eluting with a gradient of 20–40 % Et_2_O/petroleum ether (40–60 °C) to yield **30** as a racemate (119 mg, 0.23 mmol, 31 %). Separation of the two diastereomer was done via silica flash column chromatography eluting with a gradient of 10–20 % Et_2_O/cyclohexane. LC–MS: *R*_f_ 2.72 (+ESI) *m*/*z* 528.5 ([*M*+H]^+^, 28 %), 2.73 (+ESI) *m*/*z* 528.5 ([*M*+H]^+^, 51 %); ^1^H NMR (300 MHz, CDCl_3_): *δ*_H_=7.60–7.14 (10 H, m, Ph, OCH*Ph*), 6.90 (1 H, d, *J*=8 Hz, 7‐CH), 6.25 (1 H, d, *J*=8 Hz, 6‐CH), 4.71–4.53 (2 H, m, NC*H*_2_CH_2_O), 4.39 (1 H, s, OC*H*Ph), 4.15 (1 H, t, *J*=6 Hz, 4‐CH), 3.81 (2 H, t, *J*=6 Hz, C*H*_2_OBn), 3.40 (3 H, s, OC*H*_3_), 2.85–2.51 (2 H, m, 1‐CH_2_), 2.60 (1 H, d, *J*=2 Hz, CC*H*), and 2.20–1.79 (4 H, m, 2‐ and 3‐CH_2_).

### Chiral separation

***N*** **‐Benzyl‐*N*‐ethyl‐9‐(2‐fluoroethyl)‐5‐methoxy‐2,3,4,9‐tetrahydro‐1*H*‐carbazole‐4‐carboxamide (6)**. 49.8 mg of a racemic mixture of **6** was sent to Reach Separations to be separated into the two enantiomers. They provided the following methods for separation and purification. The racemate **6** was dissolved to 12 mg mL^−1^ in MeOH/CH_2_Cl_2_ (1:1) and was then purified by SFC using a Lux C1 column (21.2 mm×250 mm, 5 μm) at 40 °C and eluting with isocratic 50:50 MeOH/CO_2_ with 0.2 % *v*/*v* NH_3_ at a flow rate of 50 mL min^−1^. The injection volume was 1500 μL and peaks were detected at 223 nm. Combined fractions of each of the two enantiomers were then evaporated to near dryness using a rotary evaporator, transferred into final vessels with CH_2_Cl_2_, which was removed under a stream of compressed air at 40 °C before being stored in a vacuum oven at 40 °C and 5 mbar for 4 h to afford the two enantiomers as yellow gums. Chiral purity of the enantiomers was analysed using a Lux C1 column (4.6 mm×250 mm, 5 μm) at 40 °C and eluting with isocratic 50:50 MeOH/CO_2_ with 0.2 % *v*/*v* NH_3_ at a flow rate of 4 mL min^−1^. The injection volume was 1.0 μL and the peaks were detected at 210–400 nm. Chemical purity of the enantiomers was analysed using an Acquity BEH C_18_ column (50×2.1 mm, 1.7 μm) at 40 °C and eluting with 0–4 min gradient of 5–95 % MeCN/H_2_O with 0.1 % TFA, 4‐4.02 min gradient of 95–100 % MeCN/H_2_O with 0.1 % TFA, 4.03 min–4.5 min isocratic 100 % MeCN with 0.1 % TFA, 4.5–4.52 min gradient of 100–5 % MeCN/H_2_O with 0.1 % TFA, and 4.53–6 min isocratic of 5 % MeCN/H_2_O with 0.1 % TFA at flow rate of 0.6 mL min^−1^. The injection volume was 1.0 μL and the peak was detected at 220 nm. The final yield is 23.9 mg of enantiomer (*S*)‐**6** with 98.02 % chemical purity and in 100 % *ee*, and 22.1 mg of enantiomer (*R*)‐**6** with 100 % chemical purity and in 98.8 % *ee*.

**2‐(4‐(Benzyl(ethyl)carbamoyl)‐5‐methoxy‐1,2,3,4‐tetrahydro‐9*H*‐carbazol‐9‐yl)ethyl 4‐methylbenzenesulfonate (25)**. 162.4 mg of a racemic mixture of **25** was sent to Reach Separations to be separated into the two enantiomers. They provided the following methods for separation and purification. The racemate **25** was dissolved to 23 mg mL^−1^ in MeOH/CH_2_Cl_2_ (1:1) and was then purified by SFC using a Lux C1 column (21.2 mm×250 mm, 5 μm) at 40 °C and eluting with isocratic 50:50 MeOH/CO_2_ with 0.2 % *v*/*v* NH_3_ at a flow rate of 50 mL min^−1^. The injection volume was 1000 μL and peaks were detected at 223 nm. Combined fractions of each of the two enantiomers were then evaporated to near dryness using a rotary evaporator, transferred into final vessels with CH_2_Cl_2_, which was removed under a stream of compressed air at 40 °C before being stored in a vacuum oven at 40 °C and 5 mbar for 4 h to afford the two enantiomers as white solids. Chiral purity of the enantiomers was analysed using a Lux C1 column (4.6 mm×250 mm, 5 μm) at 40 °C and eluting with isocratic 50:50 MeOH/CO_2_ with 0.2 % *v*/*v* NH_3_ at a flow rate of 4 mL min^−1^. The injection volume was 1.0 μL and the peaks were detected at 210–400 nm. Chemical purity of the enantiomers was analysed using an Acquity BEH C_18_ column (50×2.1 mm, 1.7 μm) at 40 °C and eluting with 0–4 min gradient of 5–95 % MeCN/H_2_O with 0.1 % TFA, 4‐4.02 min gradient of 95–100 % MeCN/H_2_O with 0.1 % TFA, 4.03 min–4.5 min isocratic 100 % MeCN with 0.1 % TFA, 4.5–4.52 min gradient of 100‐5 % MeCN/H_2_O with 0.1 % TFA, and 4.53–6 min isocratic of 5 % MeCN/H_2_O with 0.1 % TFA at flow rate 0.6 mL min^−1^. The injection volume was 1.0 μL and the peak was detected at 220 nm. The final yield is 82.7 mg of enantiomer (*S*)‐**25** with 97.42 % chemical purity and in 98.2 % *ee*, and 77.1 mg of enantiomer (*R*)‐**25** with 98.75 % chemical purity and in 99.6 % *ee*.

### Determination of compound binding affinities at wild‐type and Ala147Thr TSPO

Binding affinities were measured as per our published protocol.^21^ Briefly, an Ultra‐Turrax homogeniser was used to prepare membranes from HEK cells stably transfected with wild‐type and A147T TSPO. These cells were previously validated as an in vitro model of LABs and HABs.^21^ Membranes (20 μg per well, diluted in 50 mm Tris⋅HCl, pH 7.4) were incubated with ≈*K*_d_ concentration of [^3^H]**1** (10 nm; PerkinElmer) and test compounds (0.3 nm–10 μm) at 4 °C for 90 min. Reactions were terminated by filtration through a 96‐well glass‐fibre filter plate (Millipore), and washed eight times with ice‐cold 50 mm Tris⋅HCl. Microscint 0 was added to the dry filters, and radioactivity read in a Microbeta^2^ 2450 Microplate Counter (PerkinElmer). Data were analysed using GraphPad Prism 6.0, and a four‐parameter nonlinear regression curve fit was used to calculate *K*_i_ values. Data are expressed as the mean±SEM from at least three independent experiments.

**Circular dichroism spectroscopy**. Solutions of (*S*)‐**6**, (*R*)‐**6**, and (*S*)‐**5** were prepared in concentrations of 0.1–0.2 mg mL^−1^ in acetonitrile and CD spectra were collected on an Aviv 410 instrument.

### Radiochemistry

**Radioisotope production**. No‐carrier‐added aqueous [^18^F]fluoride ion was produced on a GE PETtrace cyclotron by irradiation of a 2.3‐mL silver‐bodied water target with a 25‐mA current and a 16.5‐MeV proton beam on 95 % enriched ^18^O‐H_2_O via the nuclear ^18^O(p,n)^18^F reaction.

**Preparation of the [^18^F]KF‐Kryptofix‐222 complex**. [^18^F]Fluoride in [^18^O]H_2_O was transferred and immediately trapped on an anion‐exchange resin (Waters Sep‐Pak Accell Light QMA cartridge in the carbonate form) under vacuum. Trapped ^18^F‐fluoride was eluted from the Sep‐Pak cartridge and transferred to the reaction vessel with an eluent solution containing 0.25 % wild‐type Kryptofix‐222® solution (1 mL) in basic (0.05 % wild‐type K_2_CO_3_) aq. MeCN (75 % *v*/*v*). The solvents were evaporated in vacuo (130 mbar) with a stream of N_2_ gas at 95 °C over 5 min. Anhydrous MeCN (3×0.7 mL) was then added and the mixture was azeotropically dried in vacuo (130 mbar) with a stream of N_2_ at 95 °C.

**Manual preparation of [^18^F]6**. Fluoride was dried as described above and dissolved in MeCN (4 mL) before eluting from TRACERlab FX_FN_. The resulting solution was divided into three portions, two of which the MeCN was removed at 100 °C under a flow of N_2_ before dissolving in DMF or DMSO. Precursors **25** and **26** were dissolved to a concentration of 1 mg mL^−1^ in MeCN, DMSO and DMF to afford three solutions of each precursor. For each reaction, four HPLC vials were prepared containing precursor solution (125 μL) and [^18^F]fluoride solution (125 μL) of equivalent solvent. The vials were heated in a heating block to the desired temperature (80, 100 or 120 °C) and a single vial was removed at each time point (2, 5, 10, 20 min) and quenched with 1:1 MeCN/H_2_O (300 μL) before analyzing directly by HPLC using a gradient method: (pre‐injection) 1 min equilibration at 10:90 MeCN/H_2_O. (post‐injection) 1 min isocratic at 10:90 MeCN/H_2_O, 2 min gradient to 70:30 MeCN/H_2_O, 3 min isocratic at 70:30 MeCN/H_2_O. Flow rate: 1.5 mL min^−1^. Run time: 6 min (7 min including equilibration time). Column: ACE® UltraCore, Super C_18_, 2.5 μm, 50×4.6 mm; Serial No. A133239. A portion of the sample from 20 min was taken and used for co‐injection with reference compound **6** to confirm synthesis of desired product.

**Automated preparation and formulation of [^18^F]6**. The tosylate precursor **25** (1.2 mg, 2.94 μmol) was dissolved in anhydrous MeCN (1 mL), and the solution was added to the dry [^18^F]KF‐Kryptofix‐222 complex and heated at 100 °C for 20 min. The reaction mixture was then cooled with compressed air and diluted with anhydrous MeCN (1 mL). The crude mixture was injected into an ACE 160433 C_18_ 5 μm (100×10 mm) semipreparative reversed‐phase HPLC column. With a mobile phase of 48 % aq. MeCN at a flow rate of 5 mL min^−1^. [^18^F]**6** was collected (*t*_R_: 19.6 min) and immediately diluted with H_2_O (10 mL). The aqueous solution was passed through a C_18_ cartridge (Waters Sep‐Pak Accell Light C_18_ cartridge, prepared by washing with 2 mL of ethanol, rinsing with 10 mL of water). The cartridge was washed with H_2_O (15 mL) and the radiolabelled product was eluted with EtOH (0.5 mL). The radiolabelled product was then formulated with 0.9 % *w*/*v* aq. NaCl in a vial to afford the radiolabelled title compound.

**Quality control for [^18^F]6**. To confirm the identity of the formulated radiotracer, and for the determination of molar radioactivity and radiochemical purity, a portion of the final solution with known radioactivity was injected into a Phenomenex Luna 5 μm C_18_ (2) 100 Å (250×4.6 mm) analytical reversed‐phase HPLC column. A mobile phase of 0–3 min isocratic at 10 % aq. MeCN, 3–5 min gradient 10–80 % aq. MeCN, and 6–12 min isocratic 80 % aq. MeCN at a flow rate of 1.5 mL min^−1^ to elute [^18^F]**6** at a *t*_R_ of 9.8 min. The area of the UV absorbance peak at 220 nm corresponding to the carrier product was measured (integrated) on the HPLC chromatogram and compared with a standard curve relating mass to UV absorbance. The identity of the radiolabelled product was confirmed by HPLC co‐injection of the reference compound **6**.

### In vivo PET imaging

Male Wistar rats (*n*=2; weight: 230 and 302 g) were obtained from Charles River Laboratories, UK. The rats were housed in Techniplast 2000P IVC cages on a layer of Aspen bedding in a room with constant temperature (21±2 °C) and fixed 12 h light–dark regime (lights on at 7:00 am). Food and water were available ad libitum. After arrival, the rats were allowed to acclimatise for at least seven days. This research was regulated under the Animals (Scientific Procedures) Act 1986 Amendment Regulations 2012 following ethical review by the University of Cambridge Animal Welfare and Ethical Review Body (AWERB).

Prior to scanning, the animals were anaesthetised using isofluorane at a concentration of 5 % in O_2_, and anaesthesia was maintained at 1.5–2.5 % isofluorane in O_2_. The femoral vein was cannulated for tracer injection. Body temperature was maintained at 37 °C using an electronic heating blanket. Anaesthetised rats were placed, one per scanning session, in a microPET Focus 120 scanner (modified in house according to the reference).^22^ They were positioned on a heating mat in transaxial position with their head in the field of view. A transmission scan of 515 s with a ^68^Ge point source was made for attenuation and scatter correction of 511 keV photons. The radiotracer, formulated in saline, was injected to the rats and the emission scan was started with tracer injection. A list‐mode protocol was used with an acquisition time of 60 min.

Image reconstructions were performed using microPET Manager 2.4.1.1, ASIPro 6.7.1.2 (Siemens). Acquisition data were then Fourier re‐binned in 22 time frames (6×10 s, 4×30 s, 4×60 s, 1×180 s, 4×300 s, 3×600 s) and the data were reconstructed per timeframe employing an iterative reconstruction algorithm (ordered subsets expectation maximisation, OSEM 2D with Fourier re‐binning, four iterations, and 16 subsets). The final datasets consisted of 95 slices with a slice thickness of 0.8 mm, and an in‐plane image matrix of 128×128 pixels. Voxel size was 0.8×0.8×0.8 mm. Data sets were corrected for decay, random coincidences, scatter and attenuation.

Three‐dimensional regions of interest (ROIs) were drawn over the whole brain on an MRI template using PMOD software (version 3.2; PMOD technologies, Zurich, Switzerland). PET images were then co‐registered with this MRI template and the regions of interest transferred from MRI to PET. Whole‐brain TACs were then obtained for each of the animals. The results were expressed as dimensionless standardised uptake values (SUV=[(tissue activity)×(body weight)]/injected dose). SUVs were calculated assuming a specific gravity of 1 g mL^−1^ for brain tissue.

## Conflict of interest

*The authors declare no conflict of interest*.

## Supporting information

As a service to our authors and readers, this journal provides supporting information supplied by the authors. Such materials are peer reviewed and may be re‐organized for online delivery, but are not copy‐edited or typeset. Technical support issues arising from supporting information (other than missing files) should be addressed to the authors.

Supplementary
