# Supplementary material for: Radiosynthesis of (R,S)‐[18F]GE387: A Potential PET Radiotracer for Imaging Translocator Protein 18 kDa (TSPO) with Low Binding Sensitivity to the Human Gene Polymorphism rs6971
Source: ChemMedChem. 2019 Apr 8;14(9):982–93. doi: 10.1002/cmdc.201900023 (PMC6563049; doi:10.1002/cmdc.201900023)

CHEMMEDCHEM
Chemistry & Drug Discovery

Supporting Information

Radiosynthesis of (*R*,*S*)-[^{18}F]GE387: A Potential PET Radiotracer for Imaging Translocator Protein 18 kDa (TSPO) with Low Binding Sensitivity to the Human Gene Polymorphism rs6971

Luxi Qiao+,[a] Emily Fisher+,[a] Lindsay McMurray,[a] Selena Milicevic Sephton,*[a] Matthew Hird,[a] Nisha Kuzhuppilly-Ramakrishnan,[a] David J. Williamson,[a] Xiouyun Zhou,[a] Eryn Werry,[b] Michael Kassiou,[b] Saijinder Luthra,[c] William Trigg,[c] and Franklin I. Aigbirhio*[a]

cmdc_201900023_sm_miscellaneous_information.pdf

Supporting information: CONTENTS

FXF_N schematic	**S2**
NMR Spectra	**S3**
HPLC Data	**S20**

F_XFN schematic

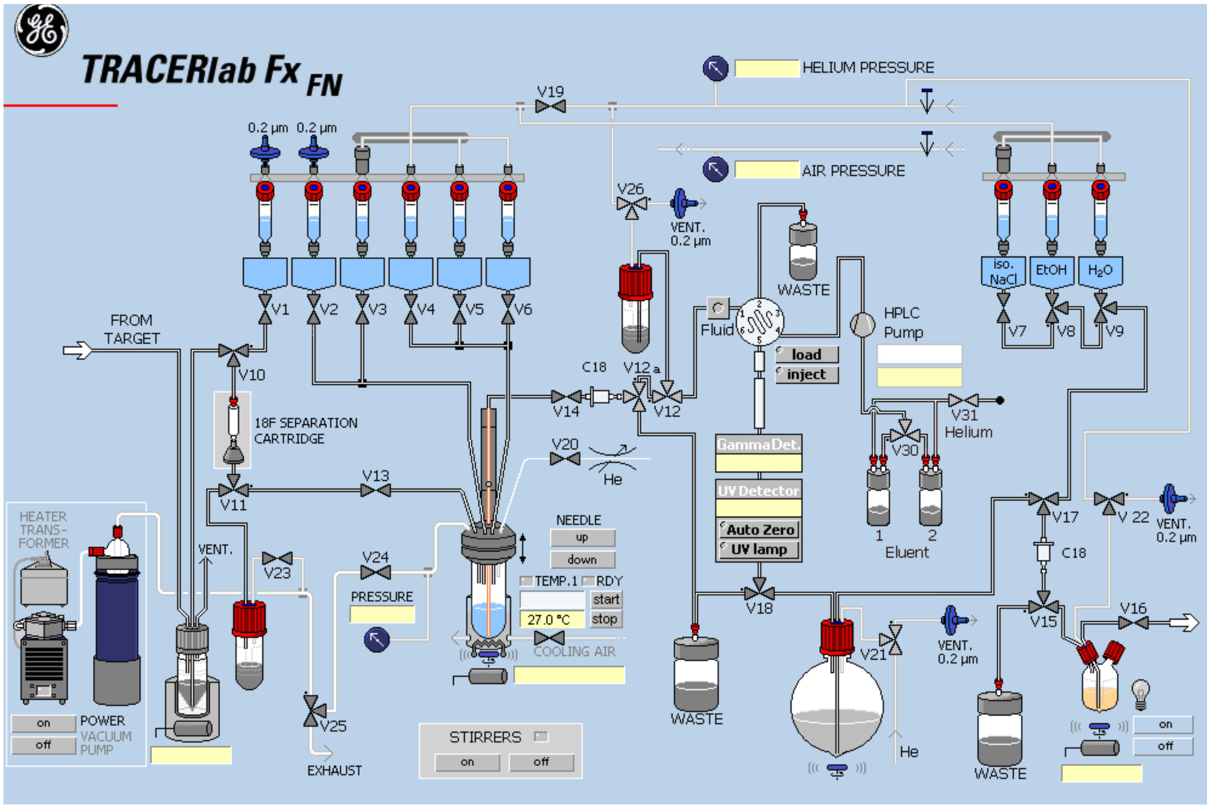

Figure S1: Schematic representation of F_XFN TRACERLab module

Supporting information: NMR Data

^{1}H NMR spectra were recorded in Fourier transform mode at the field strength specified using standard 5 mm diameter tubes. Chemical shifts in ppm is quoted relative to residual solvent signals calibrated as follows: **$CDCl_3$** δ_H (C*H*Cl_3) = 7.26 ppm; δ_C (*C*HCl_3) = 77.2 ppm. Unless otherwise stated spectra were collected at ambient temperature.

Compound	$^{1}H/^{13}C$ NMR	Page
**10**	300 MHz, $CDCl_3$	S4
**13**	300 MHz, $CDCl_3$	S5
**6**	300/75 MHz, $CDCl_3$	S6/7
**18**	300 MHz, $CDCl_3$	S8
**20**	300 MHz, $CDCl_3$	S9
**21**	300 MHz, $CDCl_3$	S10

Compound	$^{1}H/^{13}C$ NMR	Page
**25**	300/75 MHz, $CDCl_3$	S11/12
**26**	300 MHz, $CDCl_3$	S13
**27**	300 MHz, $CDCl_3$	S14
**29**	300 MHz, $CDCl_3$	S15/16
**30**	300 MHz, $CDCl_3$	S17/18

SFC data for chiral separation of **6** **S20/21**

Semi-prep HPLC data for [^{18}F]**6** **S22/23**

Chiral HPLC data for [^{18}F]**6** **S24/25**

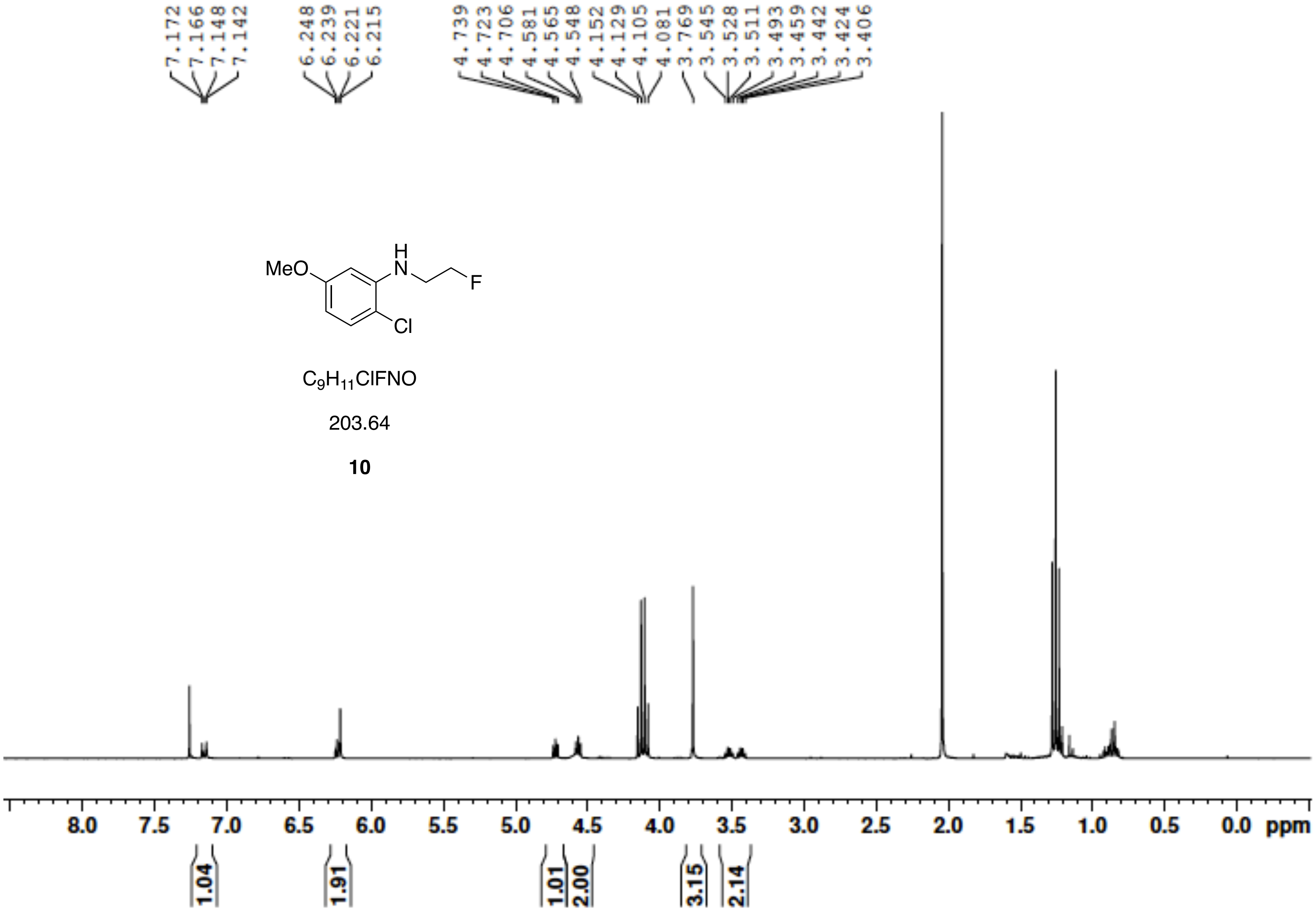
7.172
7.166
7.148
7.142
6.248
6.239
6.221
6.215
4.739
4.723
4.706
4.581
4.565
4.548
4.152
4.129
4.105
4.081
3.769
3.545
3.528
3.511
3.493
3.459
3.442
3.424
3.406
MeO
H
N
F
Cl
$C_9H_{11}ClFNO$
203.64
10
8.0 7.5 7.0 6.5 6.0 5.5 5.0 4.5 4.0 3.5 3.0 2.5 2.0 1.5 1.0 0.5 0.0 ppm
1.04
1.91
1.01
2.00
3.15
2.14

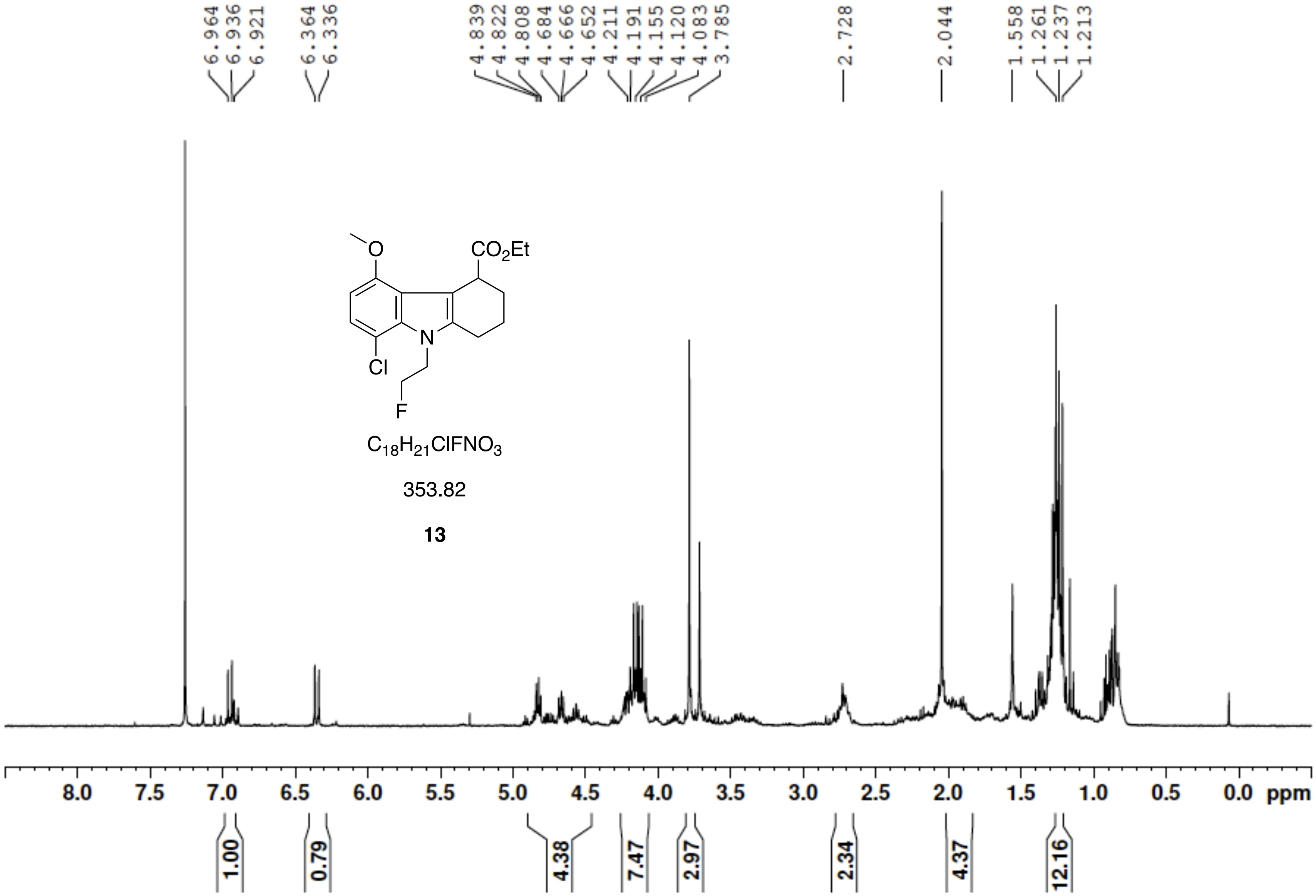
6.964
6.936
6.921
6.364
6.336
4.839
4.822
4.808
4.684
4.666
4.652
4.211
4.191
4.155
4.120
4.083
3.785
2.728
2.044
1.558
1.261
1.237
1.213
O
CO2Et
N
Cl
F
C18H21ClFNO3
353.82
13
8.0
7.5
7.0
6.5
6.0
5.5
5.0
4.5
4.0
3.5
3.0
2.5
2.0
1.5
1.0
0.5
0.0 ppm
1.00
0.79
4.38
7.47
2.97
2.34
4.37
12.16

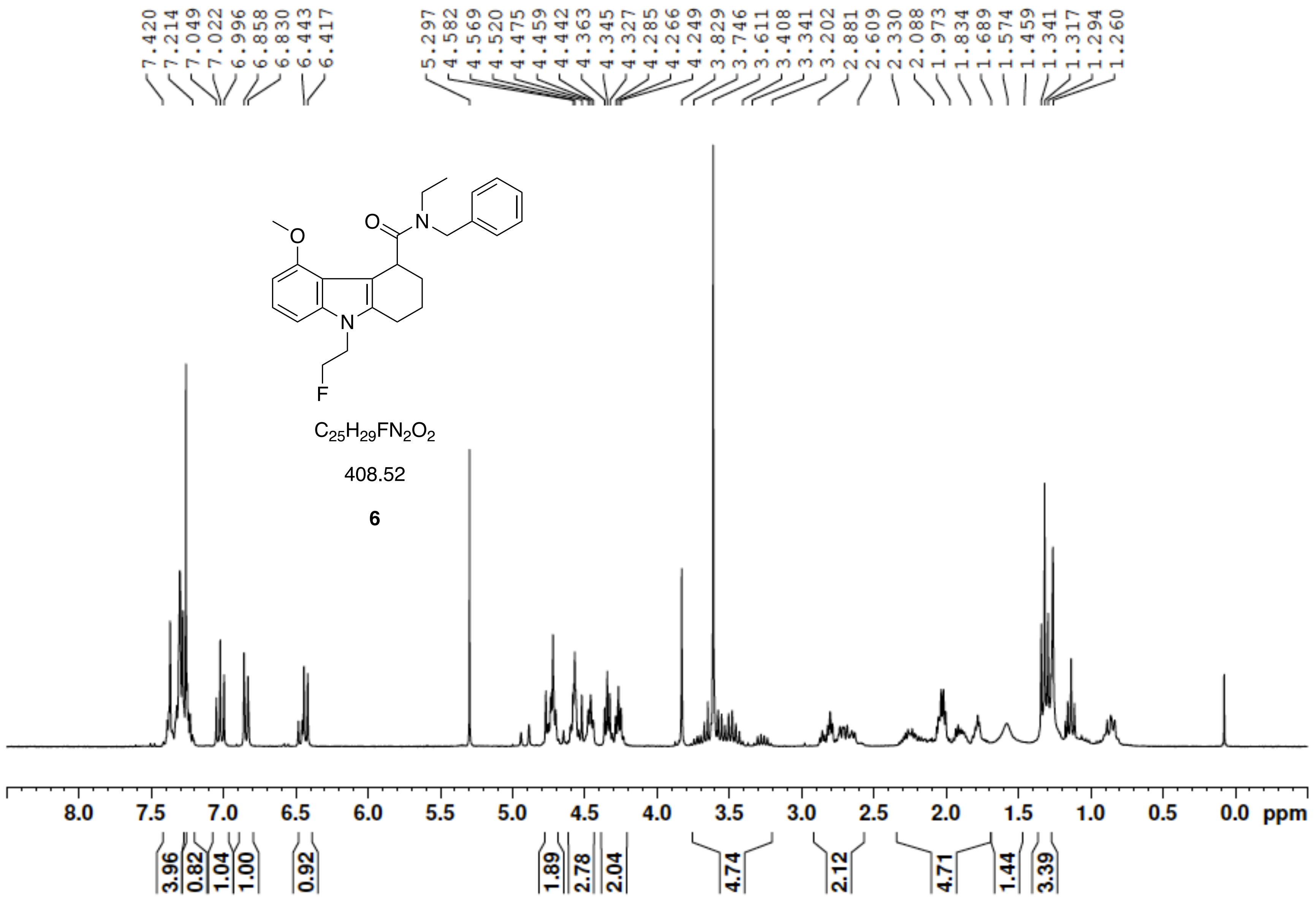

7.420
7.214
7.049
7.022
6.996
6.858
6.830
6.443
6.417
5.297
4.582
4.569
4.520
4.475
4.459
4.442
4.363
4.345
4.327
4.285
4.266
4.249
3.829
3.746
3.611
3.408
3.341
3.202
2.881
2.609
2.330
2.088
1.973
1.834
1.689
1.574
1.459
1.341
1.317
1.294
1.260
O
N
F
$C_{25}H_{29}FN_2O_2$
408.52
6
8.0
7.5
7.0
6.5
6.0
5.5
5.0
4.5
4.0
3.5
3.0
2.5
2.0
1.5
1.0
0.5
0.0
ppm
3.96
0.82
1.04
1.00
0.92
1.89
2.78
2.04
4.74
2.12
4.71
1.44
3.39

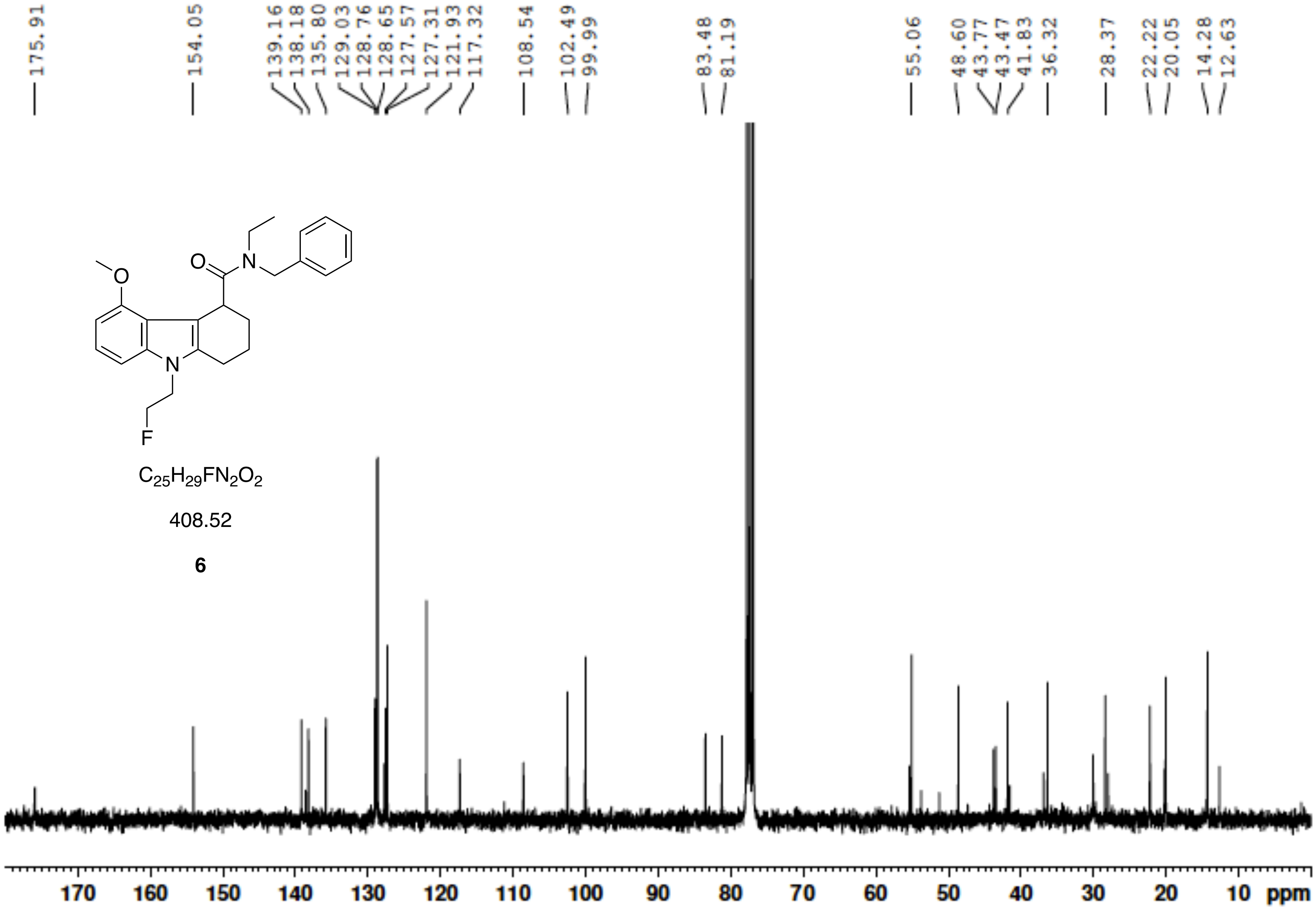

$C_{25}H_{29}FN_2O_2$

408.52

6

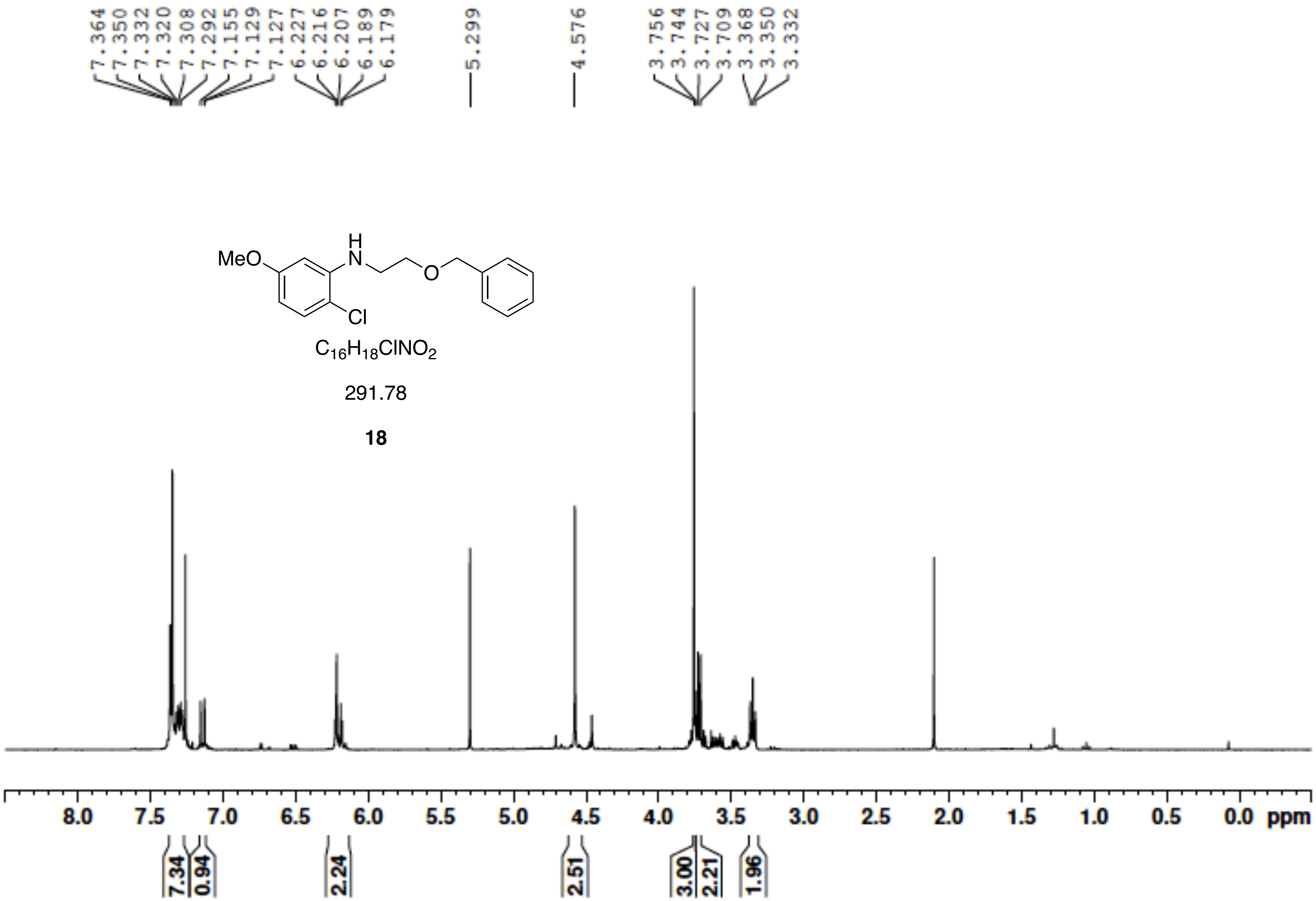
7.364
7.350
7.332
7.320
7.308
7.292
7.155
7.129
7.127
6.227
6.216
6.207
6.189
6.179
5.299
4.576
3.756
3.744
3.727
3.709
3.368
3.350
3.332
MeO
H
N
O
Cl
$C_{16}H_{18}ClNO_2$
291.78
18
8.0 7.5 7.0 6.5 6.0 5.5 5.0 4.5 4.0 3.5 3.0 2.5 2.0 1.5 1.0 0.5 0.0 ppm
7.34
0.94
2.24
2.51
3.00
2.21
1.96

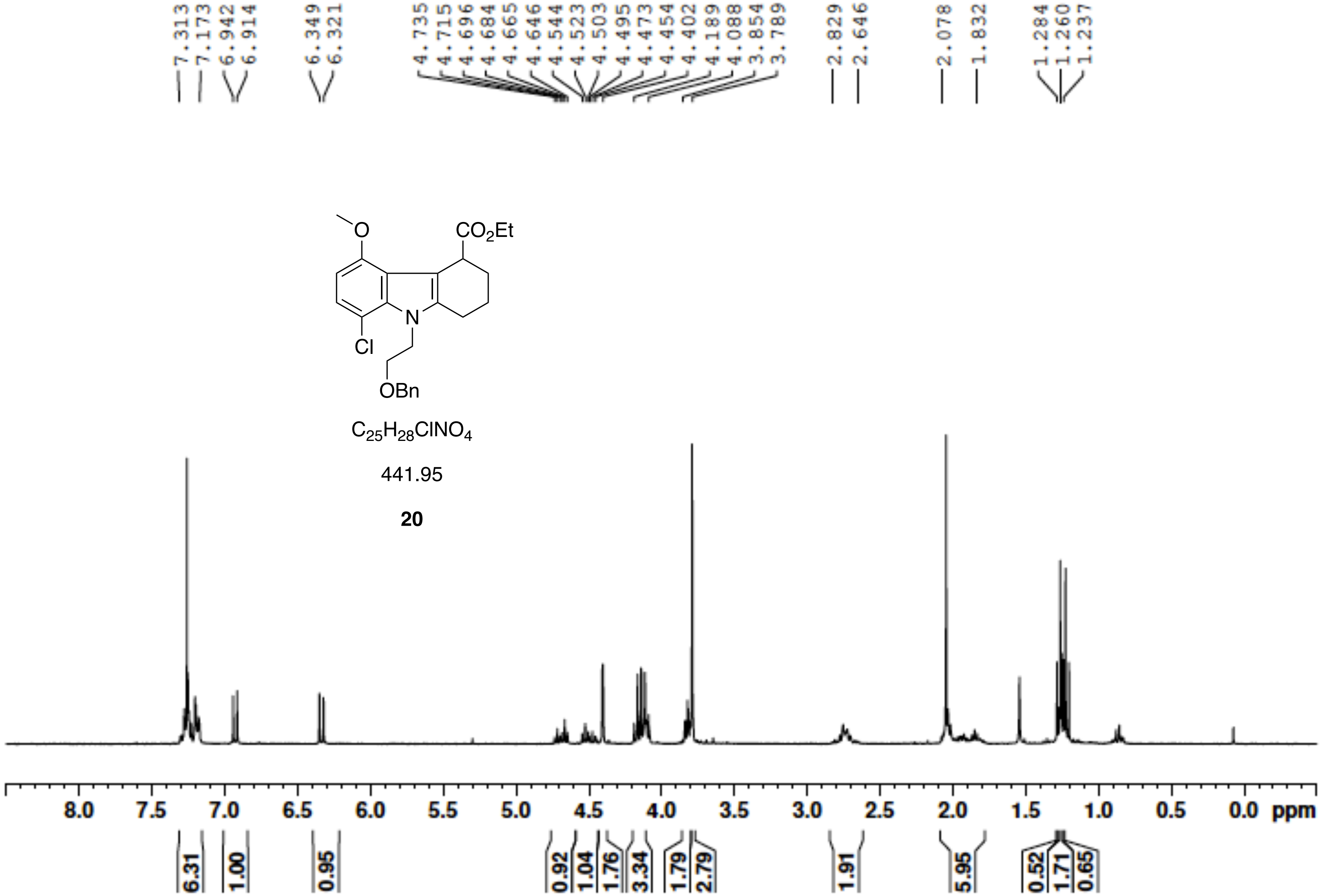
7.313
7.173
6.942
6.914
6.349
6.321
4.735
4.715
4.696
4.684
4.665
4.646
4.544
4.523
4.503
4.495
4.473
4.454
4.402
4.189
4.088
3.854
3.789
2.829
2.646
2.078
1.832
1.284
1.260
1.237
CO2Et
O
N
Cl
OBn
C25H28ClNO4
441.95
20
8.0 7.5 7.0 6.5 6.0 5.5 5.0 4.5 4.0 3.5 3.0 2.5 2.0 1.5 1.0 0.5 0.0 ppm
6.31
1.00
0.95
0.92
1.04
1.76
3.34
1.79
2.79
1.91
5.95
0.52
1.71
0.65

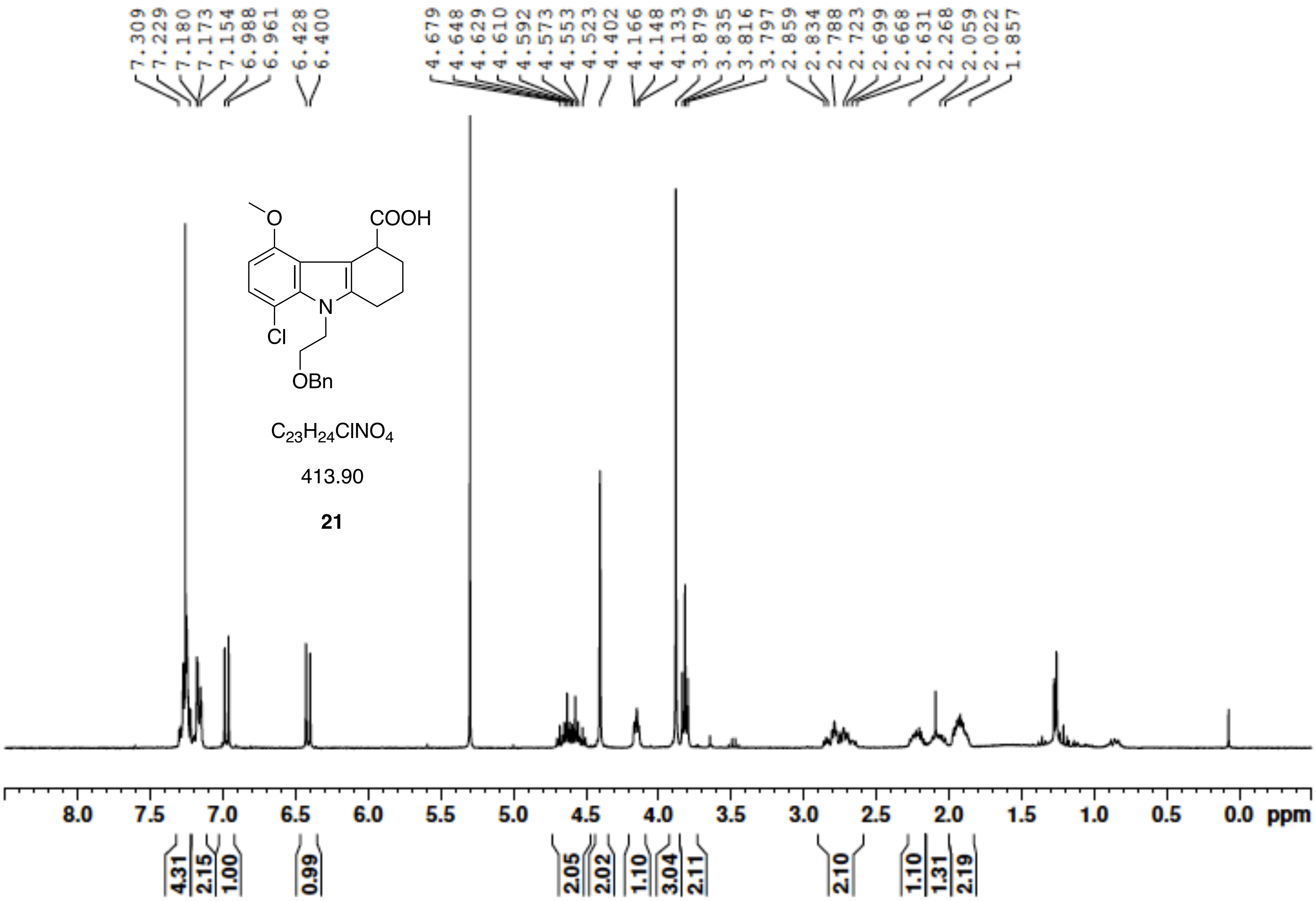
7.309
7.229
7.180
7.173
7.154
6.988
6.961
6.428
6.400
4.679
4.648
4.629
4.610
4.592
4.573
4.553
4.523
4.402
4.166
4.148
4.133
3.879
3.835
3.816
3.797
2.859
2.834
2.788
2.723
2.699
2.668
2.631
2.268
2.059
2.022
1.857
COOH
O
N
Cl
OBn
C23H24ClNO4
413.90
21
8.0 7.5 7.0 6.5 6.0 5.5 5.0 4.5 4.0 3.5 3.0 2.5 2.0 1.5 1.0 0.5 0.0 ppm
4.31
2.15
1.00
0.99
2.05
2.02
1.10
3.04
2.11
2.10
1.10
1.31
2.19

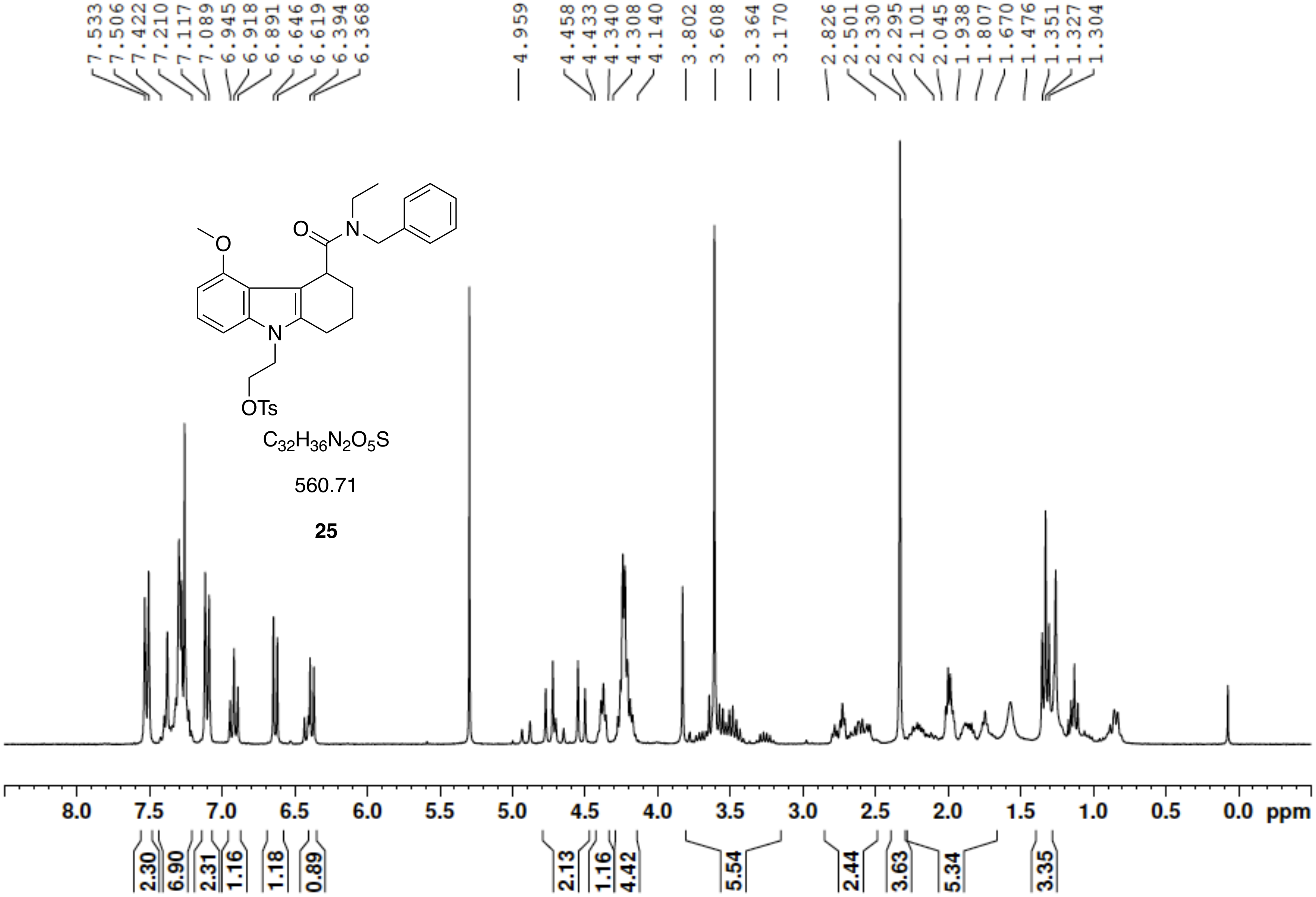

7.533
7.506
7.422
7.210
7.117
7.089
6.945
6.918
6.891
6.646
6.619
6.394
6.368
4.959
4.458
4.433
4.340
4.308
4.140
3.802
3.608
3.364
3.170
2.826
2.501
2.330
2.295
2.101
2.045
1.938
1.807
1.670
1.476
1.351
1.327
1.304
O
N
N
OTs
$C_{32}H_{36}N_2O_5S$
560.71
25
8.0 7.5 7.0 6.5 6.0 5.5 5.0 4.5 4.0 3.5 3.0 2.5 2.0 1.5 1.0 0.5 0.0 ppm
2.30
6.90
2.31
1.16
1.18
0.89
2.13
1.16
4.42
5.54
2.44
3.63
5.34
3.35

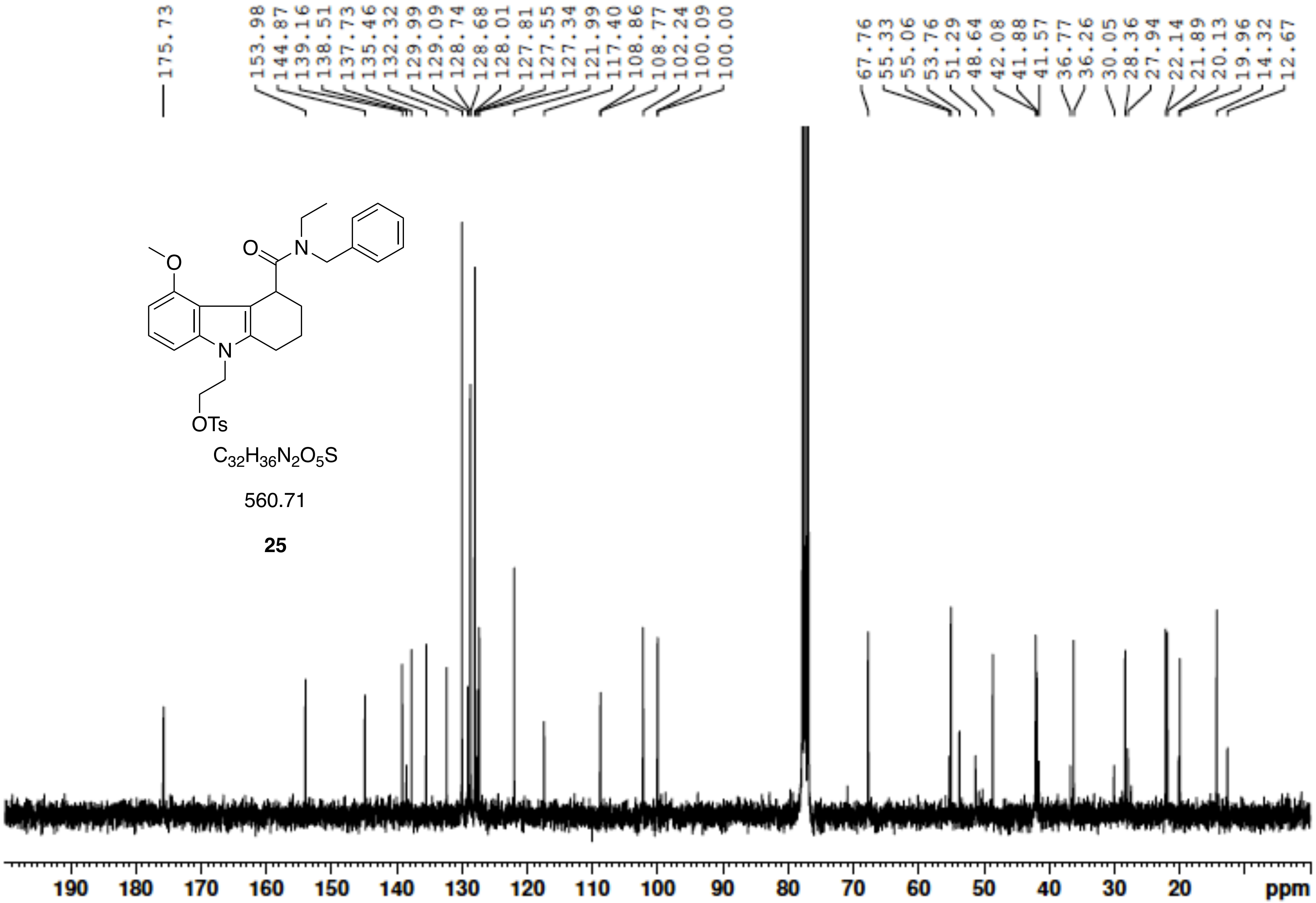

$C_{32}H_{36}N_2O_5S$

560.71

25

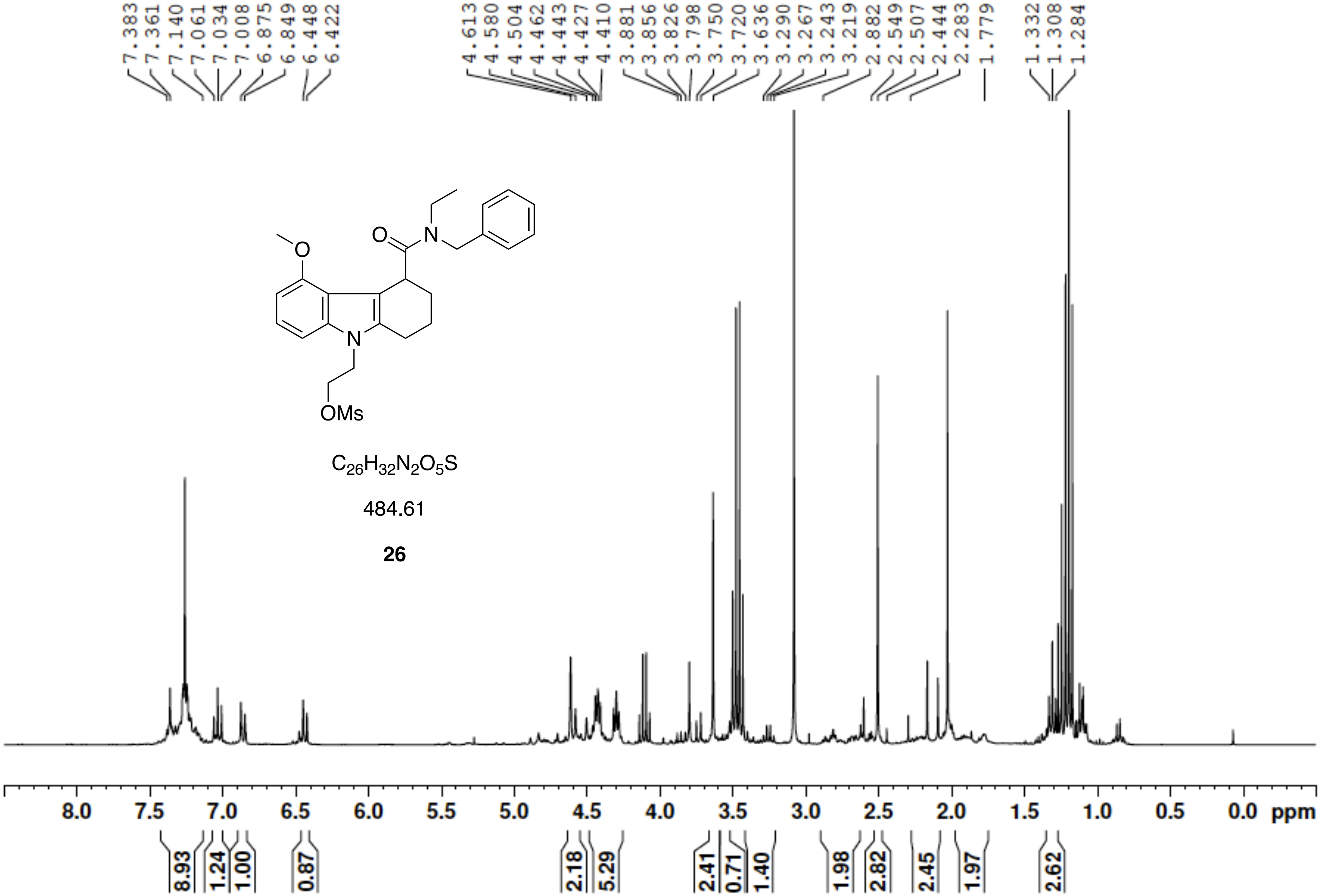
OMs
C26H32N2O5S
484.61
26
7.383
7.361
7.140
7.061
7.034
7.008
6.875
6.849
6.448
6.422
4.613
4.580
4.504
4.462
4.443
4.427
4.410
3.881
3.856
3.826
3.798
3.750
3.720
3.636
3.290
3.267
3.243
3.219
2.882
2.549
2.507
2.444
2.283
1.779
1.332
1.308
1.284
8.0
7.5
7.0
6.5
6.0
5.5
5.0
4.5
4.0
3.5
3.0
2.5
2.0
1.5
1.0
0.5
0.0
ppm
8.93
1.24
1.00
0.87
2.18
5.29
2.41
0.71
1.40
1.98
2.82
2.45
1.97
2.62

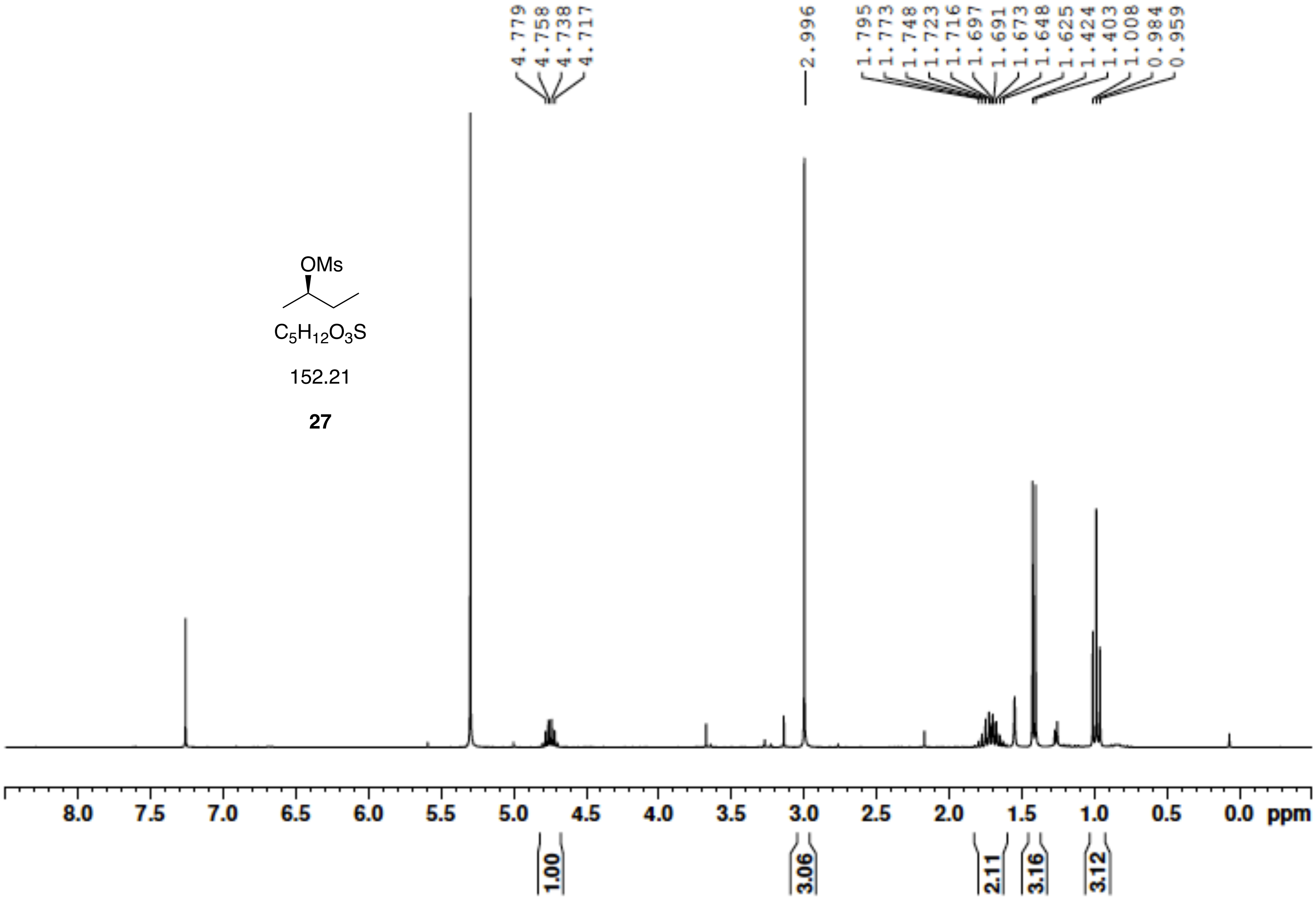
OMs
$C_5H_{12}O_3S$
152.21
4.779
4.758
4.738
4.717
2.996
1.795
1.773
1.748
1.723
1.716
1.697
1.691
1.673
1.648
1.625
1.424
1.403
1.008
0.984
0.959
8.0 7.5 7.0 6.5 6.0 5.5 5.0 4.5 4.0 3.5 3.0 2.5 2.0 1.5 1.0 0.5 0.0 ppm
1.00
3.06
2.11
3.16
3.12

27

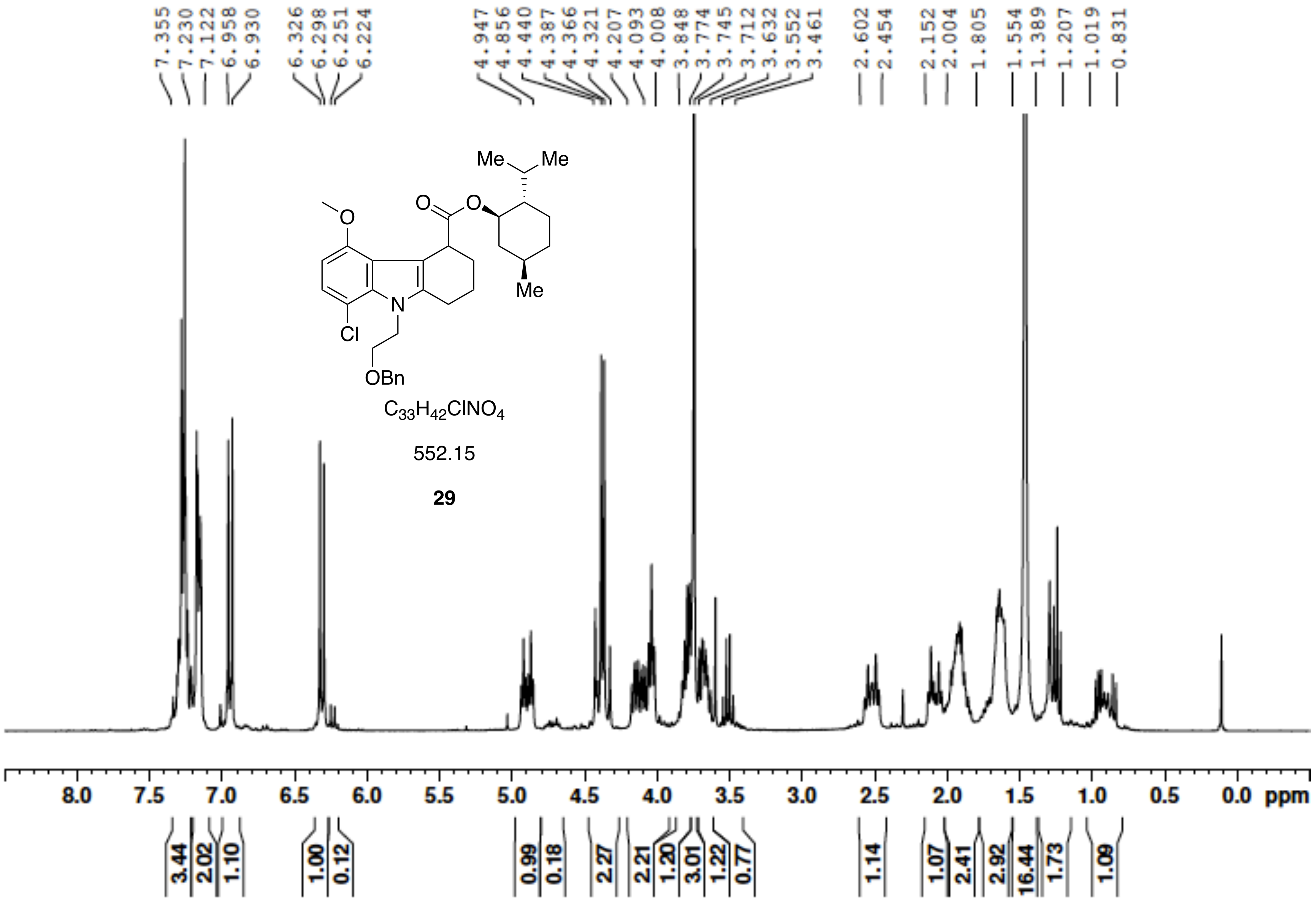
7.355
7.230
7.122
6.958
6.930
6.326
6.298
6.251
6.224
4.947
4.856
4.440
4.387
4.366
4.321
4.207
4.093
4.008
3.848
3.774
3.745
3.712
3.632
3.552
3.461
2.602
2.454
2.152
2.004
1.805
1.554
1.389
1.207
1.019
0.831
Me
Me
O
O
O
N
Cl
OBn
Me
$C_{33}H_{42}ClNO_4$
552.15
29
8.0
7.5
7.0
6.5
6.0
5.5
5.0
4.5
4.0
3.5
3.0
2.5
2.0
1.5
1.0
0.5
0.0 ppm
3.44
2.02
1.10
1.00
0.12
0.99
0.18
2.27
2.21
1.20
3.01
1.22
0.77
1.14
1.07
2.41
2.92
16.44
1.73
1.09

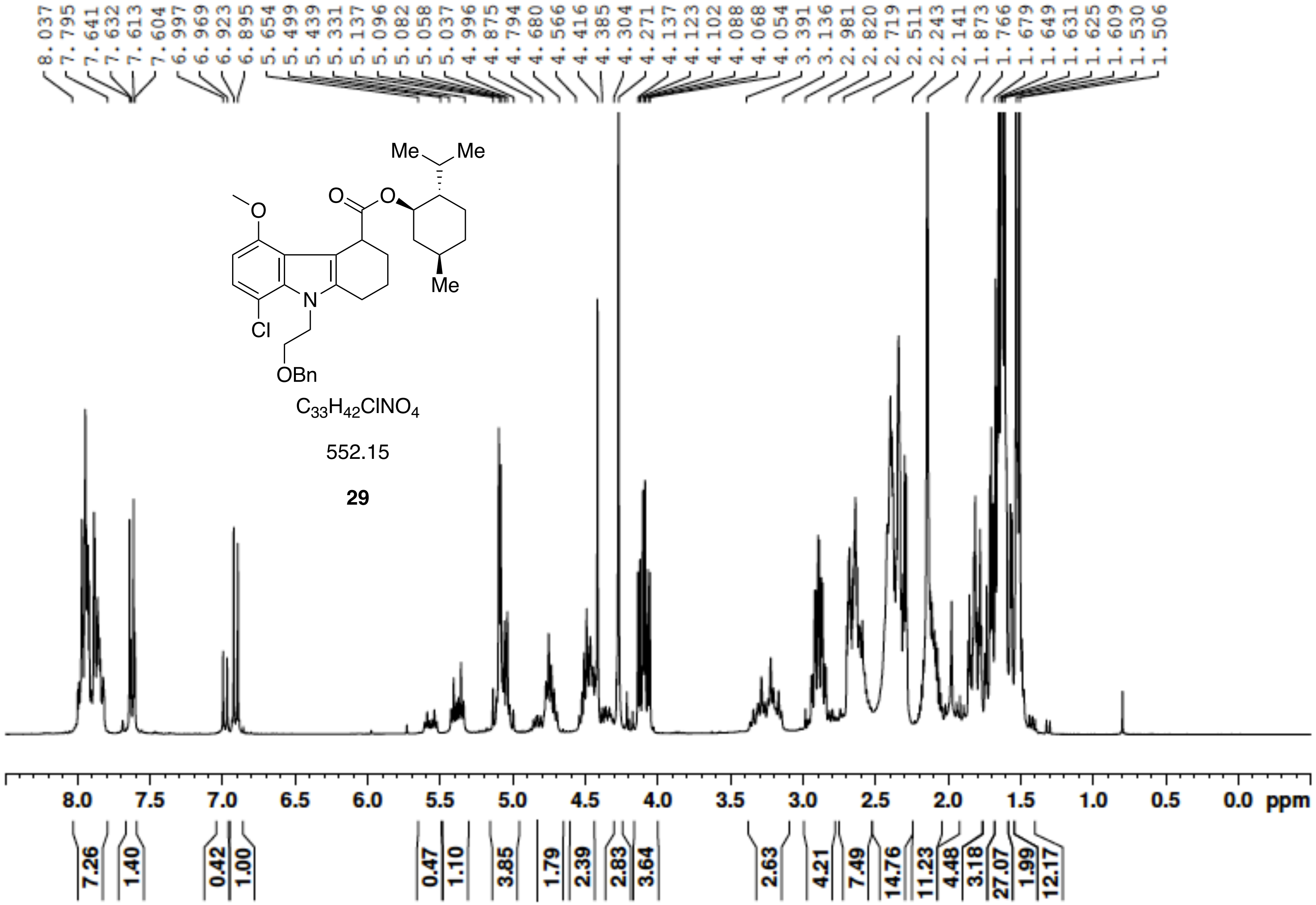
8.037 7.795 7.641 7.632 7.613 7.604 6.997 6.969 6.923 6.895 5.654 5.499 5.439 5.331 5.137 5.096 5.082 5.058 5.037 4.996 4.875 4.794 4.680 4.566 4.416 4.385 4.304 4.271 4.137 4.123 4.102 4.088 4.068 4.054 3.391 3.136 2.981 2.820 2.719 2.511 2.243 2.141 1.873 1.766 1.679 1.649 1.631 1.625 1.609 1.530 1.506
Me
Me
O
O
O
Me
N
Cl
OBn
$C_{33}H_{42}ClNO_4$
552.15
29
8.0 7.5 7.0 6.5 6.0 5.5 5.0 4.5 4.0 3.5 3.0 2.5 2.0 1.5 1.0 0.5 0.0 ppm
7.26 1.40 0.42 1.00 0.47 1.10 3.85 1.79 2.39 2.83 3.64 2.63 4.21 7.49 14.76 11.23 4.48 3.18 27.07 1.99 12.17

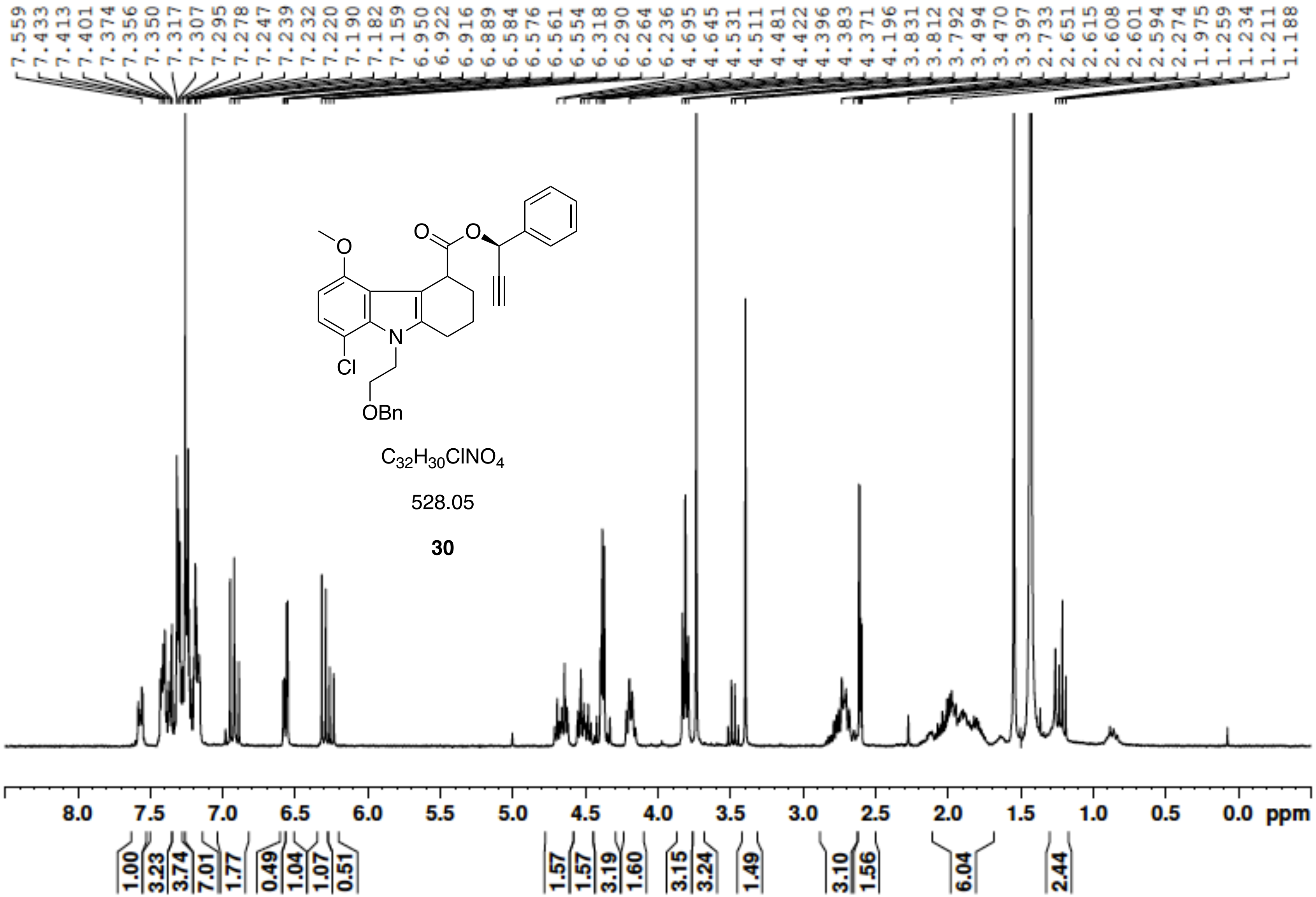

$C_{32}H_{30}ClNO_4$

528.05

30

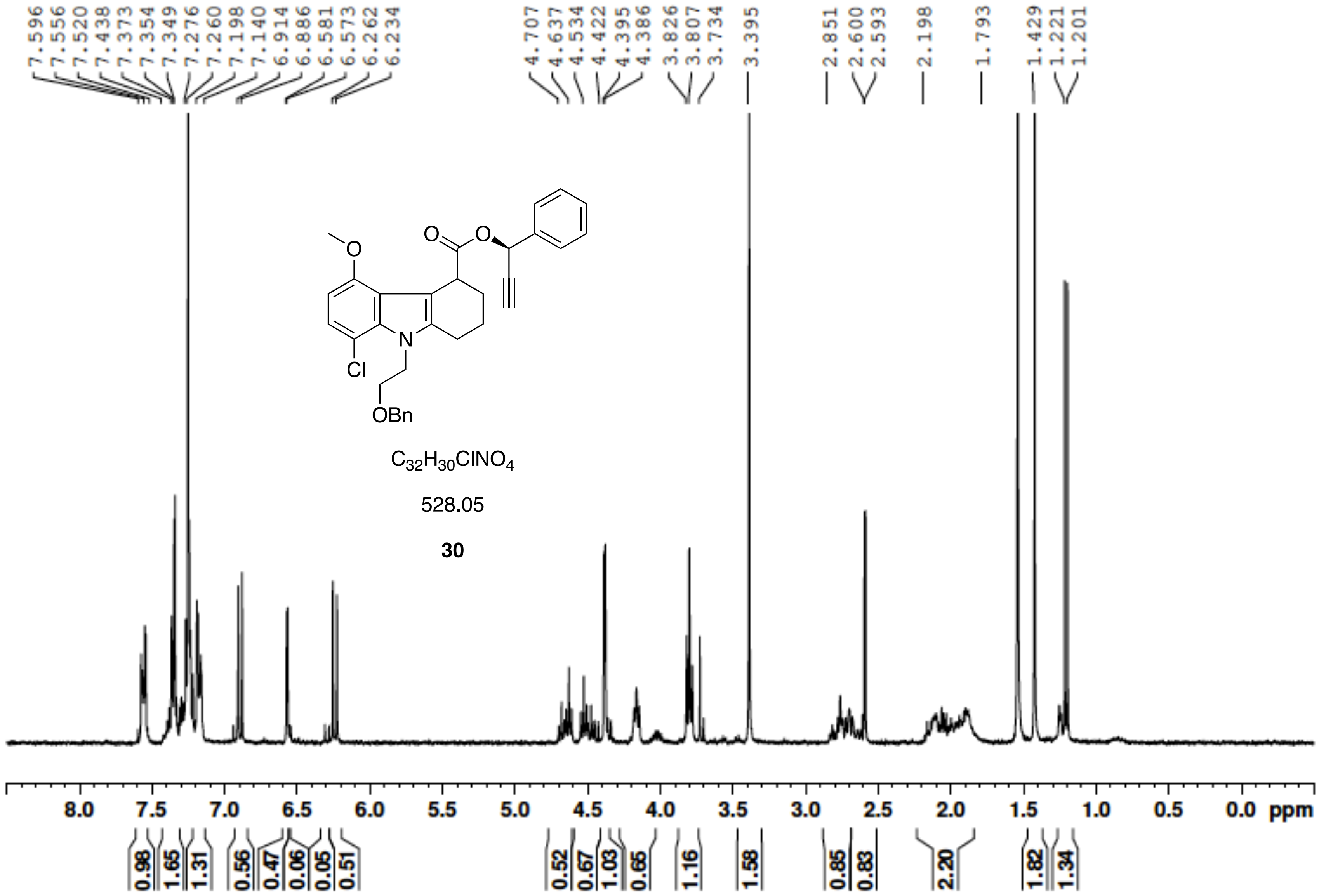
7.596
7.556
7.520
7.438
7.373
7.354
7.349
7.276
7.260
7.198
7.140
6.914
6.886
6.581
6.573
6.262
6.234
4.707
4.637
4.534
4.422
4.395
4.386
3.826
3.807
3.734
3.395
2.851
2.600
2.593
2.198
1.793
1.429
1.221
1.201
O
O
O
N
Cl
OBn
$C_{32}H_{30}ClNO_4$
528.05
30
8.0
7.5
7.0
6.5
6.0
5.5
5.0
4.5
4.0
3.5
3.0
2.5
2.0
1.5
1.0
0.5
0.0
ppm
0.98
1.65
1.31
0.56
0.47
0.06
0.05
0.51
0.52
0.67
1.03
0.65
1.16
1.58
0.85
0.83
2.20
1.82
1.34

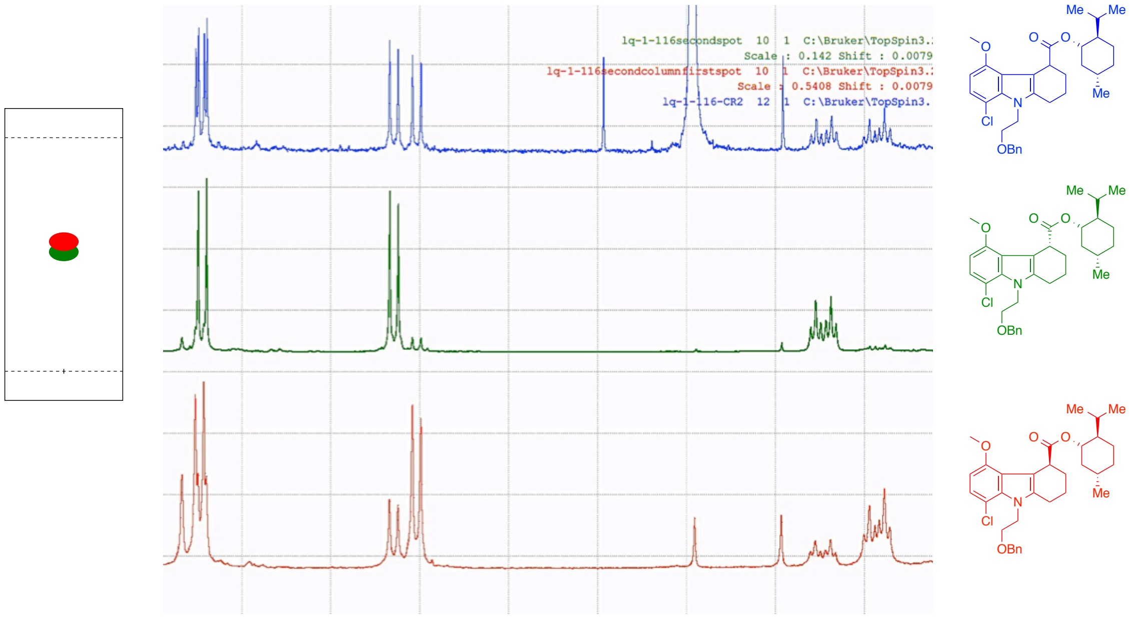

Figure S2: TLC and ^{1}H NMR evidence of the diastereomeric mixture of ester **29** (absolute stereochemistry of the diastereomers was not determined).

Chiral separation by SFC – Chromatogram of **6** (GE387)

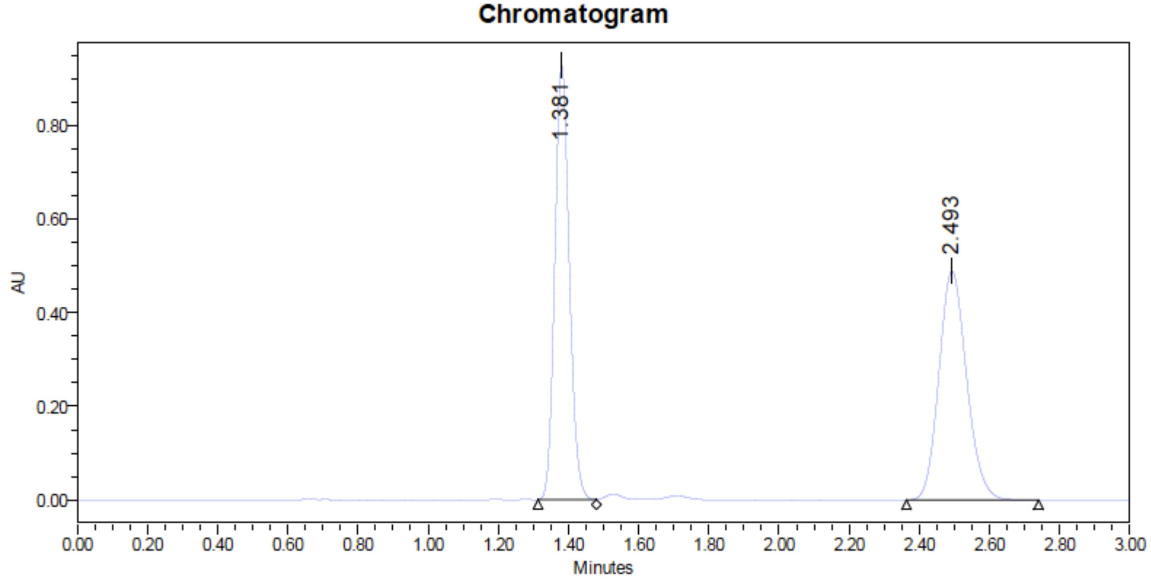

Peak Results

	Retention Time (min)	Area (µV*sec)	% Area
1	1.38	2668428	49.9
2	2.49	2682310	50.1

Chiral separation by SFC – Chromatogram of **25** (GE387 radiolabelling precursor)

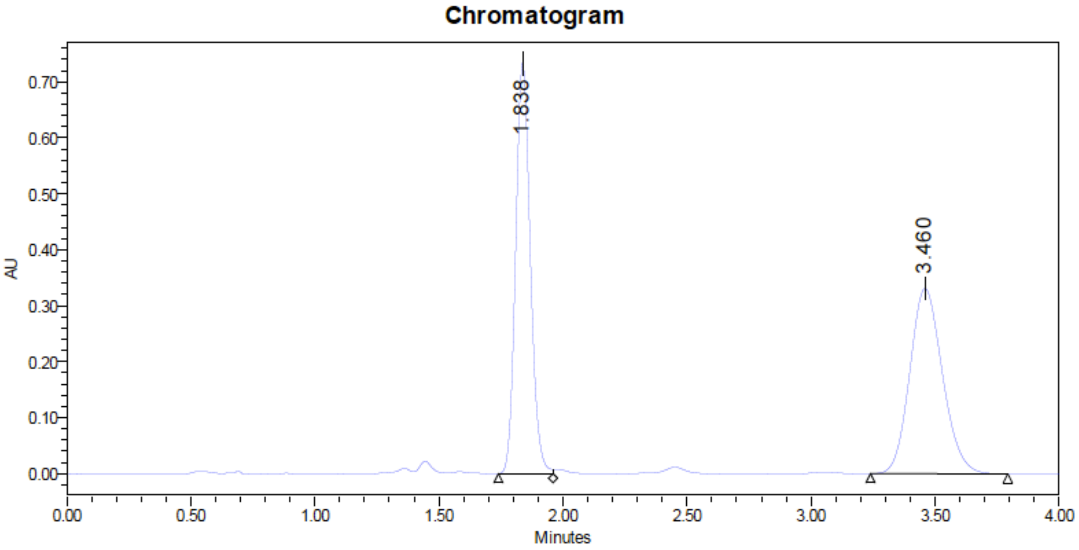

Peak Results

	Retention Time (min)	Area (µV*sec)	% Area
1	1.84	3009365	49.8
2	3.46	3030606	50.2

Gamma and UV chromatograms respectively of semi-preparative purification of a) (*S*)-[^{18}F]**6** and b) (*R*)-[^{18}F]**6**

a)

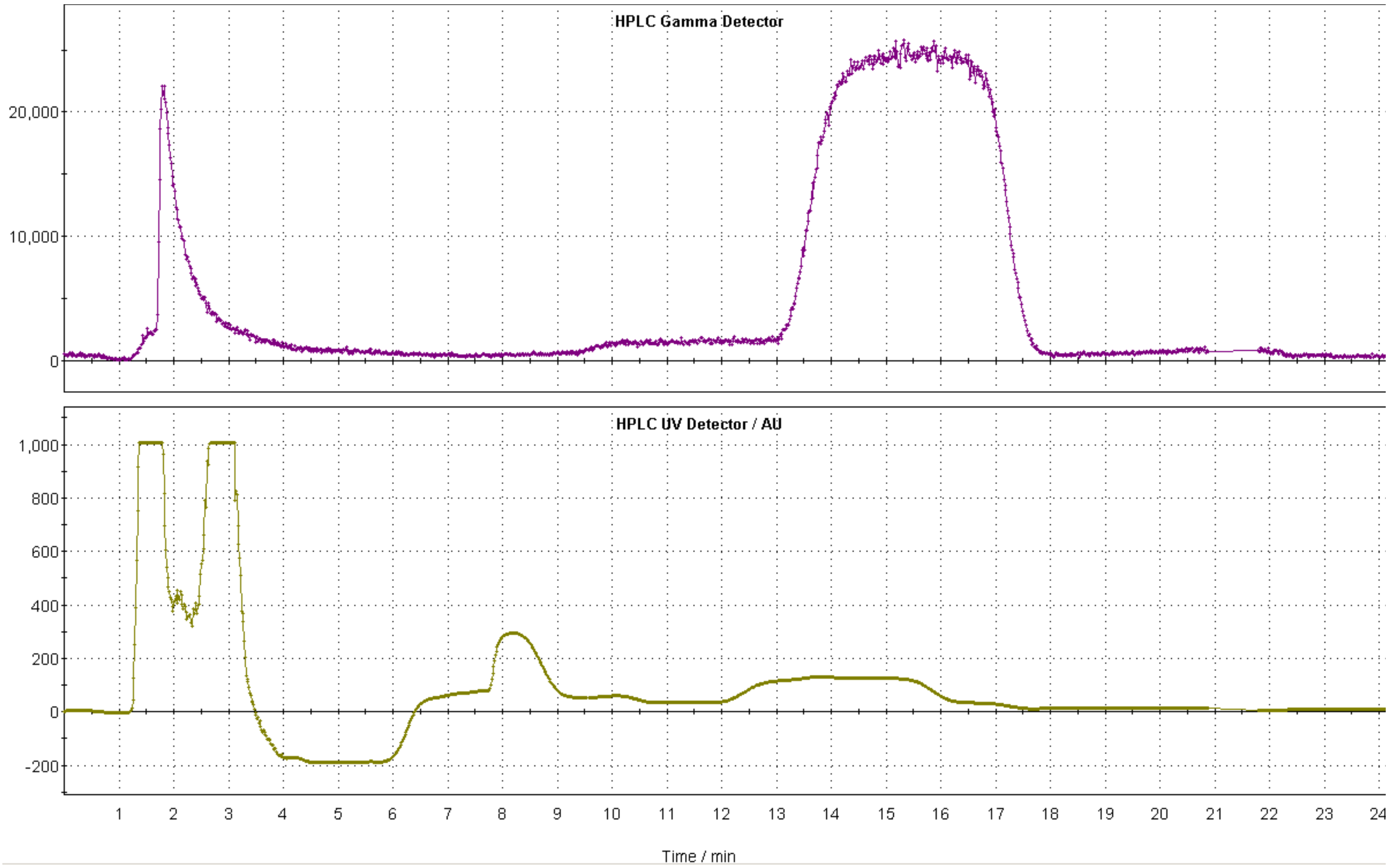

Gamma and UV chromatograms respectively of semi-preparative purification of a) (*S*)-[^{18}F]**6** and b) (*R*)-[^{18}F]**6**

b)

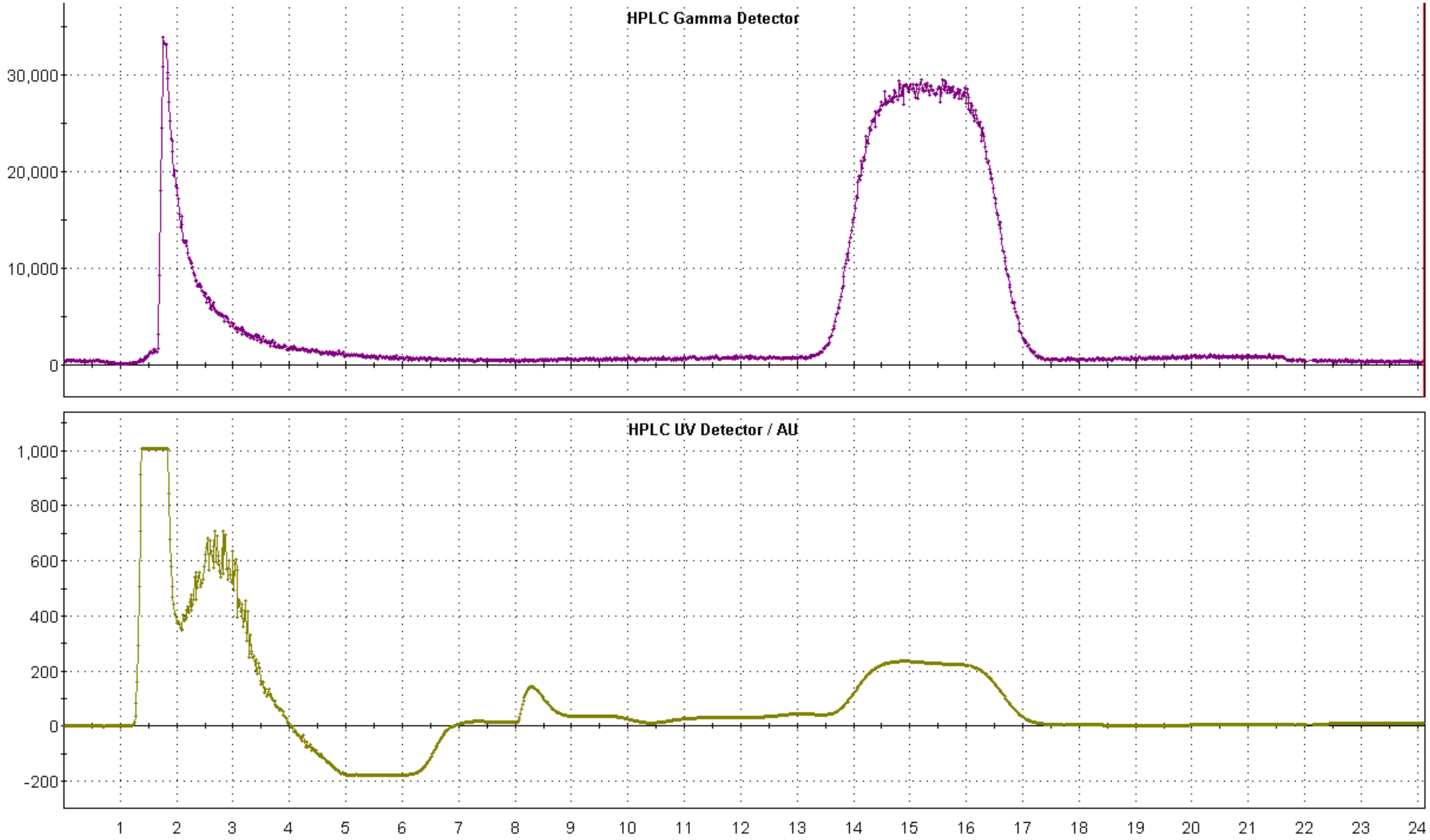

Chiral HPLC separation of a) (*S*, *R*)-**6**; b) (*S*)-**6** and c) (*R*)-**6** using Whelk-O1 (*S*, *S*) Kromasil 5 μm, 2.1x150 mm column, eluting with 40% MeOH in ^{i}PrOH at 0.3 mL/min.

a)

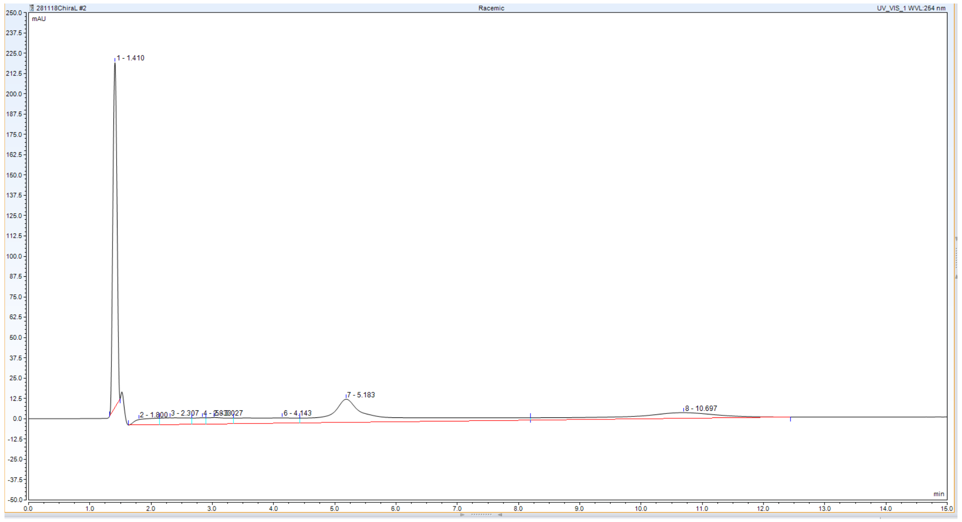

Chiral HPLC separation of a) (*S*, *R*)-**6**; b) (*S*)-**6** and c) (*R*)-**6** using Whelk-O1 (*S*, *S*) Kromasil 5 μm, 2.1x150 mm column, eluting with 40% MeOH in ^{i}PrOH at 0.3 mL/min.

b)

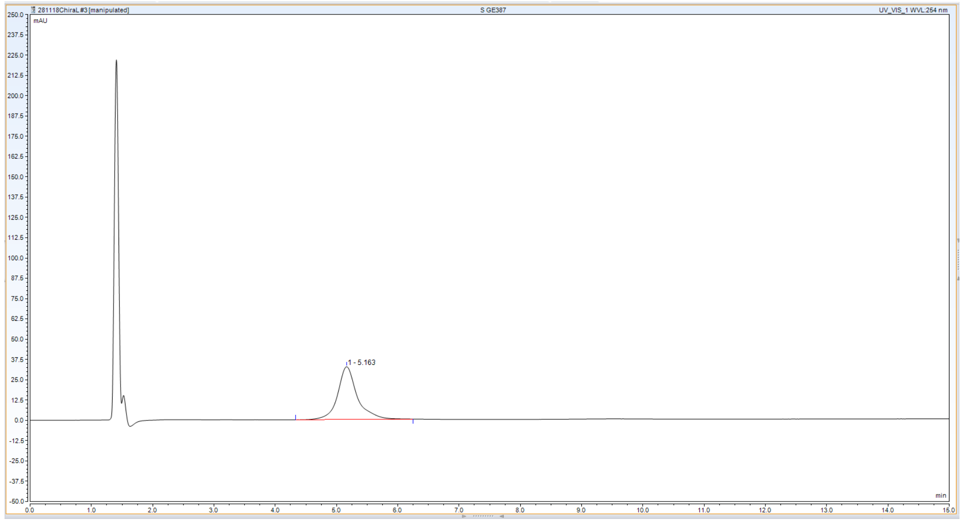

c)

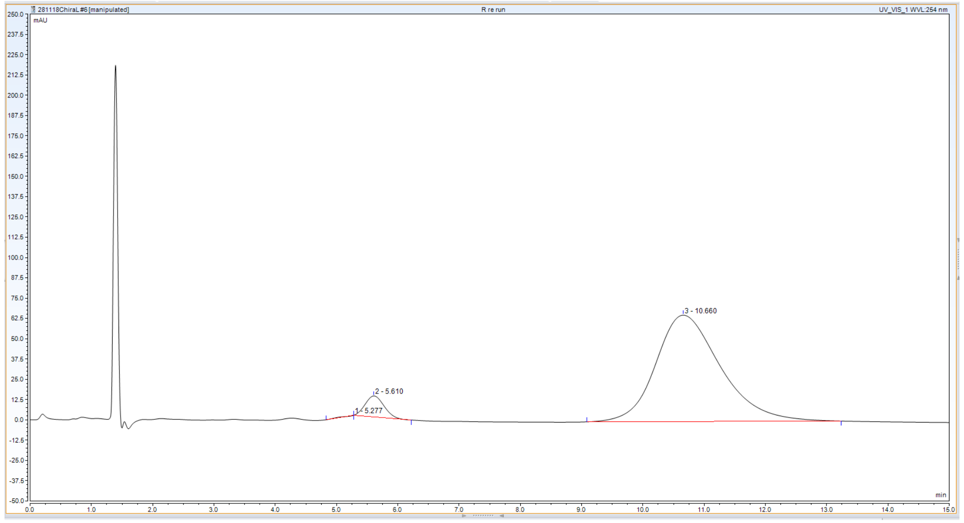

Supplement: Supplementary file 1 — Supplementary [file CMDC-14-982-s001.pdf]
